# *HEADLESS* Regulates Auxin Response and Compound Leaf Morphogenesis in *Medicago truncatula*

**DOI:** 10.3389/fpls.2019.01024

**Published:** 2019-08-16

**Authors:** Hongfeng Wang, Yiteng Xu, Limei Hong, Xue Zhang, Xiao Wang, Jing Zhang, Zhaojun Ding, Zhe Meng, Zeng-Yu Wang, Ruicai Long, Qingchuan Yang, Fanjiang Kong, Lu Han, Chuanen Zhou

**Affiliations:** ^1^The Key Laboratory of Plant Development and Environmental Adaptation Biology, Ministry of Education, School of Life Science, Shandong University, Qingdao, China; ^2^School of Life Science, Guangzhou University, Guangzhou, China; ^3^Shandong Provincial Key Laboratory of Plant Stress, Shandong Normal University, Ji’nan, China; ^4^Grassland Agri-Husbandry Research Center, Qingdao Agricultural University, Qingdao, China; ^5^Institute of Animal Sciences, Chinese Academy of Agricultural Sciences, Beijing, China

**Keywords:** *Medicago truncatula*, WUSCHEL, SAM maintenance, smooth leaf margin1 (SLM1), auxin response, compound leaf

## Abstract

WUSCHEL (WUS) is thought to be required for the establishment of the shoot stem cell niche in *Arabidopsis thaliana*. *HEADLESS* (*HDL*), a gene that encodes a WUS-related homeobox family transcription factor, is thought to be the *Medicago truncatula* ortholog of the *WUS* gene. *HDL* plays conserved roles in shoot apical meristem (SAM) and axillary meristem (AM) maintenance. *HDL* is also involved in compound leaf morphogenesis in *M. truncatula*; however, its regulatory mechanism has not yet been explored. Here, the significance of *HDL* in leaf development was investigated. Unlike *WUS* in *A. thaliana*, *HDL* was transcribed not only in the SAM and AM but also in the leaf. Both the patterning of the compound leaves and the shape of the leaf margin in *hdl* mutant were abnormal. The transcriptional profile of the gene *SLM1*, which encodes an auxin efflux carrier, was impaired and the plants’ auxin response was compromised. Further investigations revealed that *HDL* positively regulated auxin response likely through the recruitment of MtTPL/MtTPRs into the HDL repressor complex. Its participation in auxin-dependent compound leaf morphogenesis is of interest in the context of the functional conservation and neo-functionalization of the products of *WUS* orthologs.

## Introduction

The plant leaf, which varies strongly with respect to both its shape and size, is broadly classified as being either simple or compound. The former, typified by the leaves of the model dicotyledonous species *Arabidopsis thaliana*, forms a single undivided lamina, the margin of which can be smooth, serrated, or lobed. In contrast, compound leaves are composed of a number of leaflets of variable shape, each attached to the central rachis. Leaves are initiated from the shoot apical meristem (SAM) through a process of founder cell recruitment. The developmental relationship between the SAM and the leaf primordia has been a long-running topic of plant developmental biology (Arber, 1941a; Arber, 1941b; Claßen-Bockhoff, 2001).

The correct initiation of leaf and leaflet primordia and the form of the leaf margin are dependent on localized auxin concentration and PIN1 polarity (Benkova et al., 2003; Barkoulas et al., 2008; Koenig et al., 2009; Bilsborough et al., 2011; Zhou et al., 2011). Any disruption of auxin accumulation imposed by the presence of auxin transport inhibitors or mutations in auxin efflux carrier genes results in the defective development of the leaf/leaflet primordia and typically a simplification in the leaf’s form. In contrast, exogenous auxin treatment can induce ectopic leaflets and outgrowths in the leaf lamina (Barkoulas et al., 2008; Koenig et al., 2009).

Both the establishment and the maintenance of the *A. thaliana* SAM require the expression of the gene *WUSCHEL* (*WUS*), which encodes a transcription factor (Mayer et al., 1998). This enables the specification of the organizing center, a structure that determines the integrity of the stem cell niche and the maintenance of the meristem (Laux et al., 1996; Baurle and Laux, 2005; Sarkar et al., 2007; Dodsworth, 2009; Yadav et al., 2010; Yadav et al., 2011). Mutations in *WUS* result in the premature termination of both the shoot and the floral meristem (Laux et al., 1996; Mayer et al., 1998; Clark, 2001; Fletcher, 2002; Kieffer et al., 2006; Tanaka et al., 2015). In antirrhinum (*Antirrhinum majus*), the product of the *WUS* ortholog *ROA* also controls the stem cell fate in the SAM, as loss-of-function (LOF) *roa* mutants form short bushy plants, and the AMs are not maintained (Kieffer et al., 2006). In rice, the *WUS* ortholog *TAB1* (syn. *MOC3* or *OsWUS*) is inactive in both the embryo and the SAM and is not required for SAM formation. However, its presence is necessary for the initiation of the AM and for the development of tillers (Nardmann and Werr, 2006; Lu et al., 2015; Tanaka et al., 2015).

Although the participation of WUS in meristem maintenance in *A. thaliana* is well understood, the extent to which this function is conserved and/or neo-functionalized by its orthologs has been explored in only a few species. Recently, *HEADLESS* (*HDL*), the ortholog of *WUS* in *Medicago truncatula*, was identified, which played conserved roles in SAM maintenance (Meng et al., 2019). In this study, we characterized the function of *HDL* in compound leaf patterning and leaf margin formation. The molecular and genetic evidence suggested that *HDL* was involved in the maintenance of auxin homeostasis, which is critical for the leaf morphogenesis.

## Materials and Methods

### Plant Materials and Growth Conditions

*M. truncatula* ecotype R108 was used for all experiments described in this study. NF11982 (*hdl-1*; Meng et al., 2019), NF1272 (*hdl-4*; this study), *slm1-1* (Zhou et al., 2011), *mtnam-2* (Cheng et al., 2012), *mtago7-1* (Zhou et al., 2013), and *sgl1-1* (Wang et al., 2008) mutant lines were identified from a *Tnt1* retrotransposon-tagged mutant collection of *M. truncatula*. Plants were grown at 22°C day/20°C night temperature, 16 h day/8 h night photoperiod, and 70% to 80% relative humidity.

### Plasmid Construction and Plant Transformation

To generate the *HDLpro : GUS* construct, a 2366-bp promoter sequence upstream of the *HDL* start codon was amplified using primer pair HDL-Prom-F/HDL-Prom-R (**Table S2**) and transferred into the gateway destination vector pBGWFS7 (Karimi et al., 2002) vector for gene expression pattern analysis. All final binary vectors were introduced into the disarmed *Agrobacterium tumefaciens* EHA105 strain. For stable transformation, leaves of wild-type (WT) were transformed with EHA105 harboring *HDL* promoter analysis vectors (Crane et al., 2006).

### Histology, β-Glucuronidase (GUS) Staining, and Microscopy

The apical shoots of WT and *hdl-1* mutant were ﬁxed in 3% glutaraldehyde in a phosphate buffer and then dehydrated and embedded in wax. Samples were sectioned into 10-μm-thick sections using a Leica RM 2255 microtome (Leica) and then stained with toluidine blue-O (Sigma-Aldrich) for observation. For GUS staining analysis, fully expanded leaves were collected. GUS activity was histochemically detected as described previously (Zhou et al., 2011). For scanning electron microscopy (SEM), tissue samples were fixed in 3% (v/v) glutaraldehyde, dissolved in 1× PBS overnight, washed five times in 1× PBS every 10 min, dehydrated in a series of ethanol (30%, 50%, 60%, 70%, 85%, 95%, 100%, and 100% ethanol every 20 min), and then carbon dioxide (CO_2_) dried and sprayed with gold powder. The samples were observed using Tecnai G2 F20 (FEI, USA) SEM at an accelerating voltage of 15 kV.

### *In Situ* Hybridization Analysis

For RNA *in situ* hybridization, the probe fragments of 509-bp *HDL* CDS, 624-bp *SLM1* CDS, 498-bp *MtTPL* CDS, and 556-bp *MtTPR1* CDS were polymerase chain reaction (PCR) amplified using primers listed in **Table S2**. The PCR products were cloned into pGEM-T vector (Promega) and then labeled with digoxigenin-11-UTP (Roche). RNA *in situ* hybridization was performed on vegetative buds of 4-week-old WT or *hdl-1* plants as described previously (Zhou et al., 2011).

### RNA Extraction and Quantitative Real-Time PCR (qRT-PCR) Analysis

Total RNA from different organs was extracted from 6-week-old plants. Plant materials were fully ground using Tissuelyser-48 (Shanghai Jingxin). Total RNA isolation, cDNA synthesis, and qRT-PCR analysis was performed as described previously (Zhou et al., 2011). The primers used for qRT-PCR analysis are listed in **Table S2**. For all qRT-PCR analyses, three biological samples were collected.

### Phylogenetic Analysis

The protein sequences of HDL and the WUS orthologs and TPL/TPRs were used for phylogenetic analysis. The alignment of multiple protein sequences was performed using CLUSTALW online (http://www.genome.jp/tools/clustalw/). Then, the neighbor-joining phylogenetic trees in the Poisson model were constructed using the MEGA7 software suite (http://www.megasoftware.net/). The phylogenetic trees with bootstrap values from 1,000 replicates were shown.

### Yeast Two-Hybrid (Y2H) Assays and BiFC Assay

To test the protein interaction between HDL and MtTPL/MtTPRs family proteins, the CDS of HDL and MtTPL/MtTPRs genes were cloned into the pENTR/D TOPO vector using primers (**Table S2**) to generate pENTR-HDL and pENTR-MtTPL/MtTPRs, respectively. For bait and prey plasmid constructs, the DBD-MtTPL/MtTPRs and AD-HDL vectors were generated by recombination reaction between pENTR-MtTPL/MtTPRs and pDEST32 and pENTR-HDL and pDEST22. To make the mutation and deletion in WUS domain and EAR motif in HDL, mutation and deletion were introduced into pENTR-HDL using the Fast Mutagenesis System (Transgene) and then transferred into pDEST22. The bait and prey plasmids were cotransformed into yeast MAV203 strain. Yeast transformants were selected on synthetic minimal double dropout medium deficient in Trp and Leu (DDO; Clontech). Medium supplemented with SD-Leu-Trp-His-Ade (quadruple dropout, QDO; Clontech) and 1.5 mM 3-amino-1,2,4 triazole (Sigma) was used for protein interaction tests.

BiFC assays were conducted as described previously (Ou et al., 2011) with some modifications. Briefly, pENTR-HDL and pENTR-HDL-mEAR-mWUS were cloned into gateway vector pEARLEY201-YN and produced destination pEARLEY201-HDL and pEARLEY201-HDL-mEAR-mWUS vectors, whereas pENTR-MtTPL was cloned into gateway vector pEARLEY202-YC and produced destination pEARLEY202-MtTPL. The *Agrobacterium* GV3101 harboring these constructions was coinfiltrated into 4-week-old *Nicotiana benthamiana* leaves. After incubation in the dark for 24 h and then in the light for 36 h, the leaves were dissected for observation. For fluorescent imaging, a Leica LSM 780 laser scanning confocal microscope was used. The 488 nm line of an argon laser was chosen for the yellow fluorescent protein signal.

### Transient Expression Analysis in Leaves of *N. benthamiana*

For transient expression analysis, GAL4BD, GAL4BD-HDL, GAL4BD-HDL-ΔEAR, GAL4BD-HDL-ΔWUS, and GAL4BD-HDL-ΔWUS-ΔEAR were amplified from pDEST32 series vector using specific primers (**Table S2**). The products were cloned into gateway vector pK2GW7 to generate the effector constructs. The reporter and effector plasmids were transformed into *Agrobacterium* GV3101, and then different effectors were coinfiltrated with the reporter vector 35S-UAS-GUS into 4-week-old *N. benthamiana* leaves as described previously (Voinnet et al., 2015). After incubation in the dark for 24 h and then in the light for 36 h, the leaves were used for histochemical GUS staining.

### Accession Numbers

Sequence data from this article can be found in the *Medicago truncatula* Genome Project version 4.0 (http://www.medicagogenome.org/search): HDL, Medtr5g021930; SLM1, Medtr7g089360; MtAGO7, Medtr5g042590; STF, Medtr8g107210; SGL1, Medtr3g098560; MtNAM, Medtr2g078700; MtTPL, Medtr4g009840; MtTPR1, Medtr2g104140; MtTPR2, Medtr4g120900; MtTPR3, Medtr1g083700; MtTPR4, Medtr7g112460; and MtTPR5, Medtr4g114980.

## Results

### *HDL* Plays Conserved Roles in SAM and AM Maintenance

**Figure 1:**
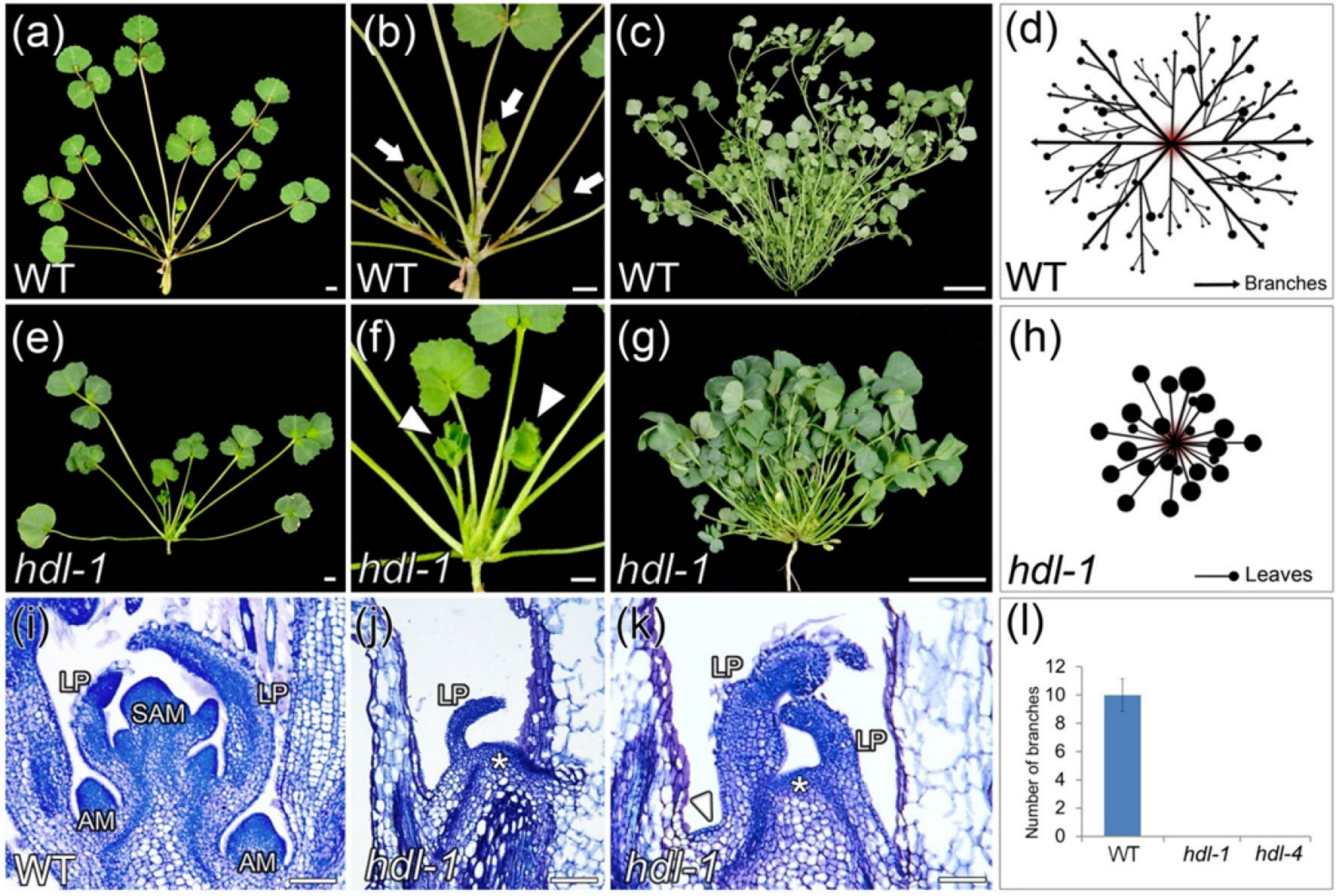
*hdl-1* mutant of *M. truncatula* shows defects in the development of branches. **(A**–**D)** 4-week-old **(A** and **B)** and 9-week-old **(C)** plants of WT. **(B)** Close view of the branches in **(A)**. **(D)** Schematic illustration of the branch arrangement in WT. Arrows indicate branches in **(B)**. **(E–H)** 4-week-old **(E** and **F)** and 9-week-old **(G)** plants of *hdl-1*. **(F)** Close view of the leaves in **(E)**. **(H)** Schematic illustration of the leaf arrangement in *hdl-1*. Arrowheads indicate leaves in **(F)**. **(I–K)** Longitudinal sections of apical shoot buds in WT **(I)** and *hdl-1* **(J** and **K)**. LP, leaflet primordium. Asterisks indicate the flattened structure in the apical position of *brl-1*. Arrowhead indicates the flattened structure between leaves. **(L)** Number of branches in 7-week-old plants of WT, *hdl-1*, and *hdl-4*. Bar, 5 mm **(A**, **B**, **E**, and **F)**, 5 cm **(C** and **G)**, and 50 µm **(I–K)**.

As for the *HDL* locus, the two mutant lines *hdl-1* and *hdl-4* were identified (**Figure S1**). A comparison of the vegetative development between the two lines showed that the developmental defects in *hdl-1* and *hdl-4* were essentially same. Compared to WT, *hdl-1* mutant exhibited an altered plant architecture (**Figures 1A–H**). Their branchless phenotype was the outcome of the emergence from the SAM of leaves but no branches (**Figures 1E–H**). The plants of both entries grew slowly, developed a bushy habit, and failed to flower. A comparison of the vegetative buds formed by WT and *hdl-1* plants showed that, within the *hdl-1* SAM, leaf primordia were initiated abnormally (**Figures 1E–K**). In addition, AM formation was completely compromised, resulting in a non-branched structure (**Figures 1J–L**).

### *HDL* Is Required for Compound Leaf Development and Leaf Margin Formation

**Figure 2:**
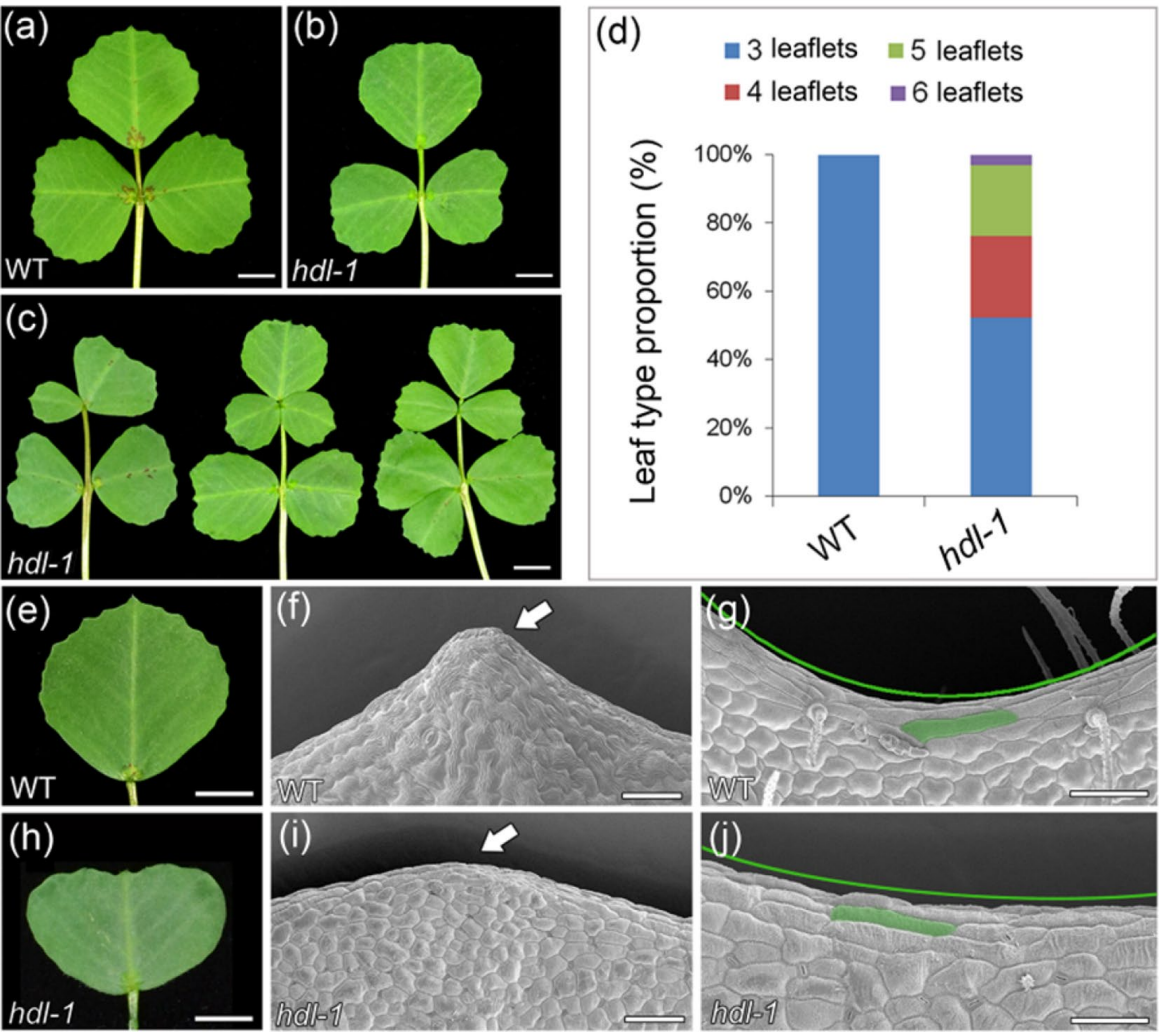
*hdl-1* mutant of *M. truncatula* shows defects in leaf development. **(A–C)** Adult leaves of WT **(A)** and *hdl-1* **(B** and **C)**. Leaflet number is significantly increased in some leaves of *hdl-1* mutant **(C)**. Bar, 5 mm. **(D)** Leaf type proportion in WT (n = 30) and *hdl-1* mutant (n = 63). **(E–J)** Development of leaf margin in WT **(E–G)** and *hdl-1* **(H–J)**. Observation of marginal cells at the teeth tips **(F** and **I)** and leaf sinus **(G** and **J)** in WT and *hdl-1* by SEM. Arrows indicate the relatively smooth leaf margin serration in *hdl-1* **(I)** compared to WT **(F)**. Green lines indicate the less pronounced sinus in *hdl-1* **(J)** compared to WT **(G)**. Representative marginal cells are marked in green color in WT **(G)** and *hdl-1* **(J)**. Bar, 5 mm **(E** and **H)** and 50 µm **(F**, **G**, **I**, and **J)**.

The WT adult leaf is composed of a terminal leaflet plus two lateral leaflets (**Figure 2A**), a morphology that differed markedly from that of the *hdl-1* mutant (**Figures 2B, C**), in which half of the adult leaves produced between one and three ectopic leaflets (**Figure 2D**). This altered compound leaf patterning was similar to that shown by the *slm1* mutant (Zhou et al., 2011). Whereas WT leaves form a strongly serrated margin (**Figures 2E–G**), the margin of those formed by the *hdl-1* mutant were relatively smooth (**Figures 2H–J**). Elongated marginal cells were present in both WT and *hdl-1* plants (**Figures 2G, J**), showing that the mutation did not influence their development. Moreover, *HDL* is also involved in stipule development. The inspection of SEM micrographs showed that leaf primordia were continuously initiated from the periphery of the SAM in WT plants (Wang et al., 2008). At stage 5, a single terminal leaflet primordium, two lateral leaflet primordia, and two stipule primordia were evident, complete with primordial trichomes on the abaxial surface of the leaf primordia (**Figure S2a**). Leaf primordia could be formed in the mutant, but their development was defective as the formation of stipule and trichomes was delayed (**Figure S2b**). Moreover, stipule size was reduced in the mutant and its stipule teeth were less sharp compared to that of WT (**Figure S2c and d**).

### *HDL* Is Expressed in Leaf Primordia and Leaf Marginal Serrations

**Figure 3:**
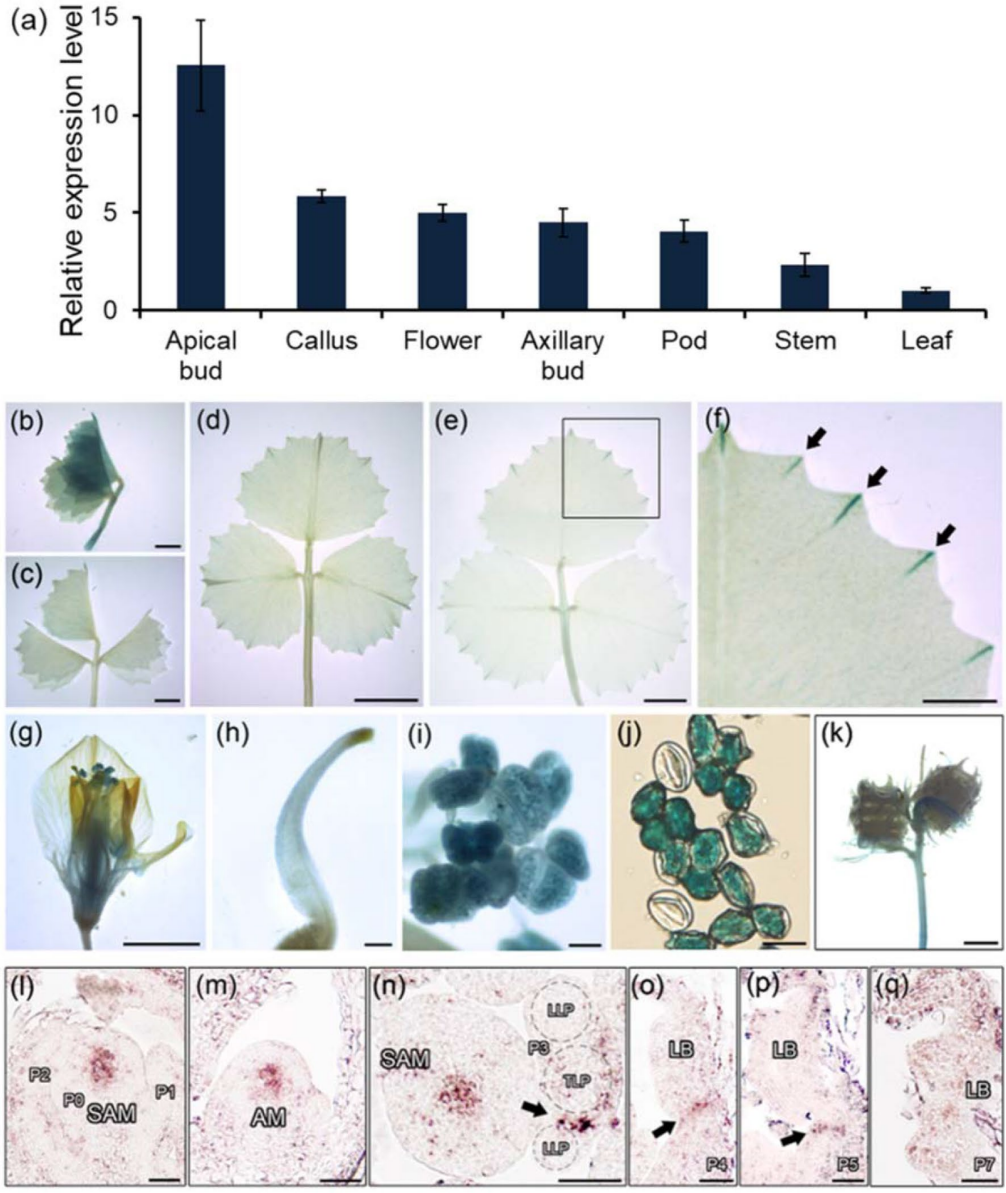
Expression patterns of *HDL* in *M. truncatula*. **(A)** Relative expression level of *HDL* in different plant organs. Three biological replicates were performed. **(B–K)** Promoter-GUS fusion studies of *HDL* expression in transgenic plants. GUS histochemical staining was detected in unexpanded leaf **(B** and **C)**, fully expanded leaf **(D** and **E)**, leaf margin serrations **(F)**, flower **(G)**, stigma **(H)**, anther **(I)**, pollen **(J)**, and seed pods **(K)**. **(F)** Close view of GUS staining of leaf margin (empty box). Arrows indicate the tips of leaf serrations. Bar, 2 mm **(B** and **C)**, 5 mm **(D–G** and **K)**, 50 µm **(H** and **I)**, and 200 µm **(J)**. **(L–Q)** RNA *in situ* hybridization analysis of *HDL* mRNA in WT. Longitudinal and transverse sections of the SAM **(L** and **N)**, longitudinal section of AM **(M)**, and longitudinal and transverse sections of leaf primordia **(N–Q)** at different developmental stages were shown. Arrows indicate the signals. P, leaf primordium; TLP, terminal leaflet primordium; LLP, lateral leaflet primordium; LB, leaf blade. Bar, 50 µm.

A qRT-PCR assay showed that *HDL* was strongly transcribed in vegetative shoot buds, axillary buds, flowers, and callus but only at a relatively low level in leaves (**Figure 3A**). When a transgene comprising the *GUS* reporter gene driven by the *HDL* promoter (*HDLpro : GUS*) was introduced into WT *M. truncatula*, the strongest levels of GUS activity were observed in the leaf buds (**Figure 3B**; **Figure S4**). Although the GUS signal was weak in the leaf lamina as a whole (**Figures 3C, D**), it was considerable at the tips of the leaf margin serrations during the course of leaf development (**Figures 3E, F**) as confirmed by qRT-PCR (**Figure S5**). GUS activity was also observed in the basal portions of flowers, stigmas, anthers, pollen grains, and immature pods (**Figure 3G–K**). A finer level of spatial and temporal resolution was achieved using RNA *in situ* hybridization (**Figures 3L–Q**; **Figure S3**); this showed that *HDL* was transcribed abundantly in the central domain of both the SAM and the AM (**Figures 3L, M**) but not during the early stages (P0, P1, or P2) of leaf primordium development (**Figure 3L**). Transverse sections taken at the P3 stage confirmed that *HDL* was transcribed in the junction between the terminal leaflet and the lateral leaflet primordia (**Figure 3N**). At the P4 and P5 stages, *HDL* transcription was concentrated in the central region of the leaf primordium (**Figures 3O, P**), whereas at P7 a low level of transcription was detectable in the leaf margins (**Figure 3Q**). The conclusion was that *HDL* likely functions not only in SAM/AM maintenance but also in the process of compound leaf development.

### *SLM1* Transcription Differs Between WT and *hdl-1* Mutant Plants

**Figure 4:**
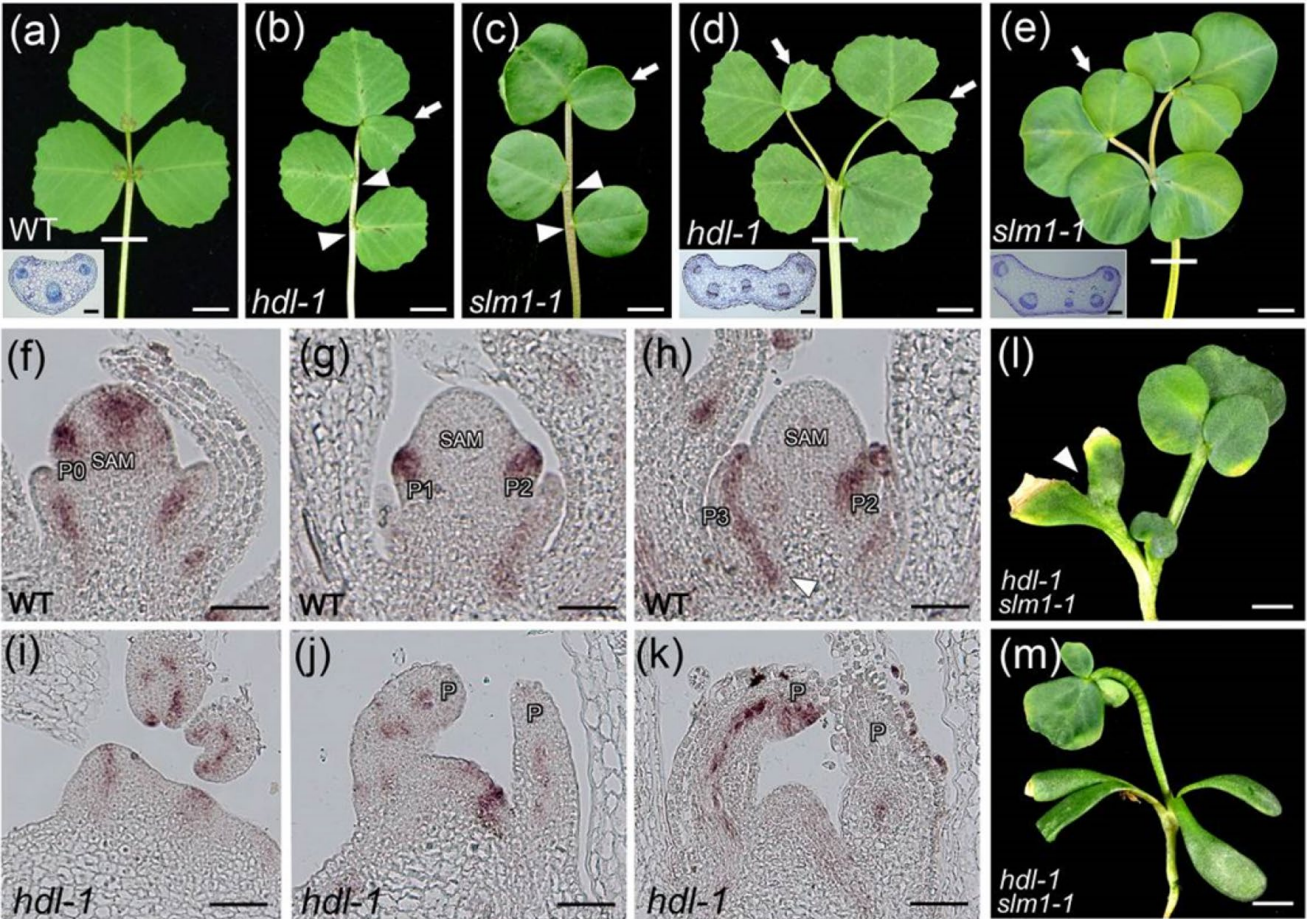
Developmental defects in *hdl* resemble those in *slm1*. **(A–C)** Compared to WT **(A)**, *hdl-1* **(B)** and *slm1-1* **(C)** mutants exhibit similar defects of leaf pattern. Arrowheads indicate asymmetric lateral leaflets. Arrows indicate ectopic terminal leaflets. Transverse section of petiole in WT is shown in the inset **(A)**. The sectioning region is shown by white line. Bar, 5 mm. (**D** and **E**) Petiole fusion can be observed in both *hdl-1* **(D)** and *slm1-1* **(E)**. Transverse sections of petioles in *hdl-1* **(D)** and *slm1-1* **(E)** are shown in the insets. White lines indicate sectioning regions. Arrows indicate ectopic terminal leaflets. Bar, 5 mm. **(F–K)** RNA *in situ* hybridization analysis of *SLM1* mRNA in the longitudinal sections of the SAM in WT **(F–H)** and *hdl-1* **(I–K)**. Arrowhead indicates provascular trace in **(H)**. Bar, 50 µm. **(L** and **M)** Phenotype of *hdl-1 slm1-1* double mutant. Arrowhead indicates fused cotyledons in **(L)**. Note that quadruple cotyledons are developed in double mutant **(M)**. Bar, 5 mm.

*SLM1* is known to encode an auxin efflux carrier protein and is an ortholog of *A. thaliana PIN1* (Zhou et al., 2011). The adult leaves of both mutants were similarly defective: in some cases, ectopic leaflets were formed, some of the lateral leaflets were asymmetric, and petioles sometimes appeared fused (**Figures 4A–E**; **Figure S6a**). The dimensions (length, width, and length/width ratio) of both the terminal and lateral leaflets formed by the *hdl-1* mutant were comparable to those formed by the *slm1-1* mutant (**Table S1**). To test the hypothesis that the defective leaf developmental shown by the *hdl-1* mutant involved a disruption in the auxin/SLM1 module, a qRT-PCR-based assay was used to contrast the transcription of *SLM1* in WT and *hdl-1* plants. This experiment showed that the abundance of transcript generated from *SLM1* and other *MtPIN* genes was unaffected by the *HDL* mutation (**Figure S6b**). However, when the comparison was based on RNA *in situ* hybridization at the early stages of leaf development, it became clear that, in WT plants, *SLM1* transcription occurred in the leaf primordia at P0, the developing leaf primordia, and the provascular trace (**Figures 4F–H**). In *hld-1* mutant, the expression level of *SLM1* was decreased, especially in SAM and leaf primordia at P0 (**Figure 4I**). The expression of *SLM1* in leaf primordia was disturbed in some cases (**Figures 4J–K**). Plants of the double mutant *hdl-1 slm1-1* were weak and dwarfed and featured both ectopic and fused cotyledons, and the small number of leaves they developed exhibited clustered leaflets (**Figures 4L, M**; **Figure S7**). The suggestion was that *HDL* and *SLM1* act synergistically during leaf initiation and compound leaf patterning.

### LOF of *HDL* Compromises the Auxin Response During the Formation of the Leaf Margin

**Figure 5:**
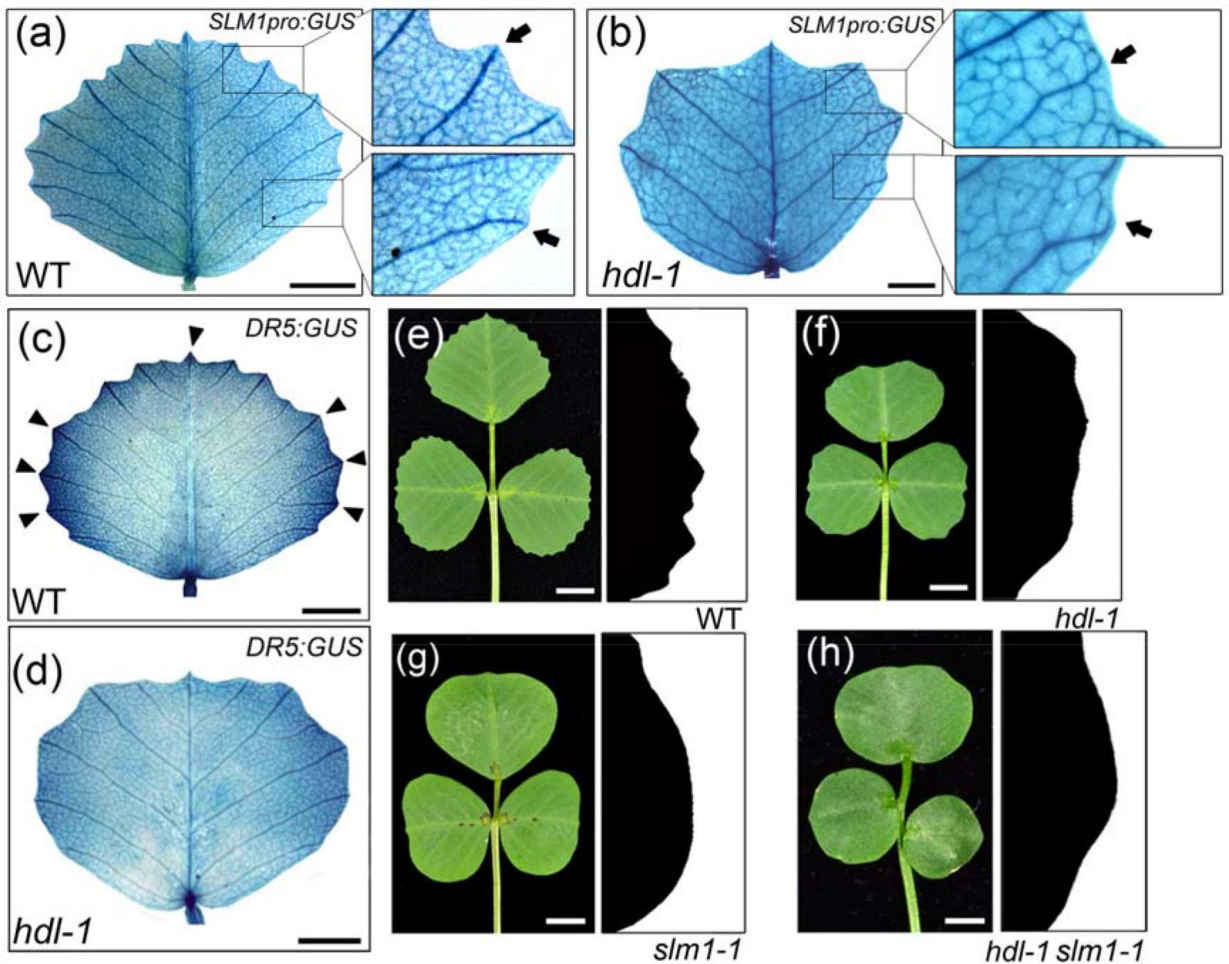
LOF of *HDL* results in the repression of auxin response in leaf margin formation. **(A** and **B)** Expression pattern of *SLM1* in fully expanded leaflet of WT **(A)** and *hdl-1* **(B)** as determined by the detection of *SLM1pro:GUS* activity. Close views of leaf margin serrations are shown in empty boxes. Arrows indicate lateral veins, which terminate at the marginal serrations in WT **(A)** but not in *hdl-1* **(B)**. Bar, 5 mm. **(C** and **D)** *DR5:GUS* expression in the fully expanded terminal leaflet of WT **(C)** and *hdl-1* **(D)**. Arrowheads mark auxin accumulation at the tip of serrations. Bar, 5 mm. **(E–H)** Leaf margin phenotype of WT **(E)**, *hdl-1* **(F)**, *slm1-1* **(G)**, and *hdl-1 slm1-1* **(H)**. Bar, 5 mm.

It is known that the auxin/*SLM1* module is a determinant of leaf margin development through its generation of local auxin activity gradients (Zhou et al., 2013). When a transgene comprising *GUS* driven by the native *SLM1* promoter (*SLM1pro:GUS*; Zhou et al., 2011) was introduced into a WT background, the resulting pattern of GUS activity revealed that the promoter activity was concentrated in the vasculature associated with serrations (**Figure 5A**). However, in *hdl-1* mutants carrying *SLM1pro:GUS*, reporter gene expression was detected in free-ending veins developed at the distal end of lateral veins (**Figure 5B**). These observations implied an alteration in auxin accumulation along the leaf margin as mediated by *SLM1*. To contrast the auxin responsiveness of WT and *hdl-1* mutant plants, the *DR5:GUS* transgenes (Zhou et al., 2011) were introduced respectively into both backgrounds. The observation was that GUS activity was decreased along the leaf margin, which was taken to imply that auxin responsiveness was repressed in the *hdl-1* mutant (**Figures 5C, D**). Like the leaves of the *slm1-1* mutant (Zhou et al., 2011), those formed by the *hdl-1*/*slm1-1* double mutant lacked marginal serration (**Figures 5E–H**), which suggested that *HDL* regulates leaf margin formation in an auxin/*SLM1*-dependent manner.

### HDL Mainly Acts as a Transcriptional Repressor

**Figure 6:**
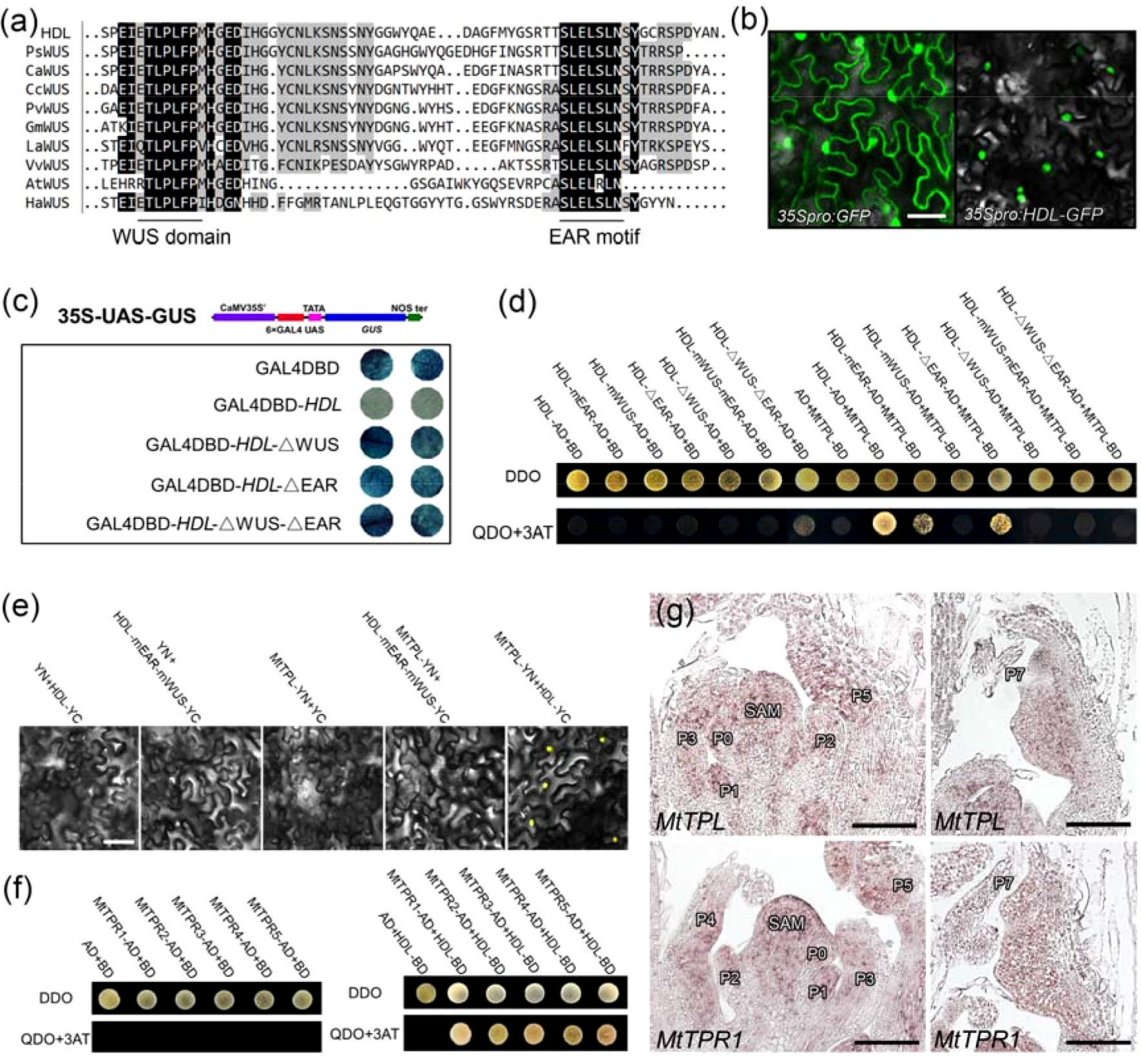
Physically interaction between HDL and MtTPL/MtTPRs. **(A)** Multiple amino acid sequence alignment of the C-terminal region containing WUS domain and EAR motif in different species.**(B)** Subcellular protein localization of HDL. HDL was localized in the nucleus. Free GFP as a control. Bar, 50 µm. **(C)** *HDL* mainly acts as a transcriptional repressor. The transcription activity of *HDL* was tested in tobacco leaves using a GAL4/UAS-based system. **(D)** WUS domain and EAR motif are required for interaction between HDL and MtTPL. Interaction was examined by yeast growth on QDO (SD-Leu-Trp-His-Ade) medium. Data are representative of three replicates. **(E)** BiFC showing the interaction between HDL and MtTPL in tobacco cells. Interaction between HDL-mEAR-mWUS and MtTPL is absent. **(F)** HDL interacts with all the MtTPR proteins. **(G)** Expression patterns of *MtTPL* and *MtTPR1* in WT. Longitudinal sections of SAM and leaf primordia are shown. Bar, 50 µm.

*HDL* encoded a protein of 302 amino acids that contained a homeodomain (residues 28–96) in the N-terminal region, a WUS domain (ETLPLFPM; between residues 240 and 247), and an EAR motif (SLELSLN; residues 285–291) in the C-terminal region (**Figure 6A**; **Figure S8**), suggesting that *HDL* may function as a transcriptional repressor. To verify this hypothesis, the construct *35S:HDL-GFP* was transiently expressed in tobacco leaves. The HDL-GFP fusion protein was localized to the nucleus (**Figure 6B**), supporting its roles as a putative transcription factor. To determine the possible transcriptional activity, a reporter construct 35S-UAS-GUS was generated. The GUS gene was driven by a synthetic promoter that contained six copies of GAL4 binding site (6×UAS) driven by a CaMV 35S promoter. Then, HDL was fused with the GAL4 DNA-binding domain (DBD) to generate GAL4DBD-HDL, which was cotransformed with 35S-UAS-GUS into tobacco leaves (**Figure 6C**). Compared to control (GAL4DBD), the GUS staining signal was severely attenuated when 35S-UAS-GUS was cotransformed with GAL4DBD-HDL (**Figure 6C**), supporting that HDL is a transcriptional repressor. To test whether the WUS domain and EAR motif in HDL were responsible for the repression activity of HDL, the HDL-ΔWUS (WUS domain deleted), HDL-ΔEAR (EAR motif deleted), and HDL-ΔWUS-ΔEAR (both WUS domain and EAR motif deleted) were fused with the GAL4 DBD and cotransformed with 35S-UAS-GUS, respectively. The results showed that these constructs could not repress GUS expression, compared to GAL4DBD-HDL (**Figure 6C**), indicating that both WUS domain and EAR motif are required for the repression activities of HDL.

### HDL Physically Interacts With the MtTPL Protein *via* Its WUS Domain and EAR Motif

It has been shown that WUS interacts with the two members of the TPL/TPR family of corepressors to function as a repressor (Kieffer et al., 2006; Causier et al., 2012). Phylogenetic analysis showed that there is one TPL (MtTPL) protein and five TPR (MtTPR1-5) proteins in *M. truncatula* (**Figure S9**). To understand the potential regulatory mechanism of *HDL*, we performed Y2H assay to examine the interaction between HDL and MtTPL. The results showed that HDL could interact with MtTPL *in vitro* (**Figure 6D**). As HDL protein contained WUS domain and EAR motif, we next test which domain and motif in HDL are responsible for the interaction with MtTPL. The interaction was completely abolished by the mutation of two Leu residues in the WUS domain (HDL-mWUS) or the deletion of WUS domain (HDL-ΔWUS; **Figure 6D**), indicating that the WUS domain is likely to be important for the interaction between HDL and MtTPL. The mutation of two Leu residues in EAR motif (HDL-mEAR) or the deletion of EAR motif (HDL-ΔEAR) somewhat reduced its interaction with MtTPL (**Figure 6D**), suggesting that the EAR motif is essential for the interaction with MtTPL. Furthermore, the combined mutation or deletion in both the WUS domain and the EAR motif in HDL also abolished the interaction with MtTPL (**Figure 6D**), which was confirmed by BiFC assay (**Figure 6E**). In addition, HDL could interact with all of the five MtTPR proteins (**Figure 6F**). To investigate whether HDL functions with MtTPL/MtTPR in leaves, spatial localization of *MtTPL*/*MtTPR1* was detected by *in situ* hybridization. The results showed that *MtTPL* and *MtTPR1* were expressed not only in SAM but also in young and old leaf primordia (**Figure 6G**), which overlapped with the expression domain of *HDL*. Taken together, these data demonstrate that HDL functions as a transcriptional repressor by recruiting the MtTPL/MtTPRs to form the complex for developmental regulation.

### Genetic Interactions Between *HDL* and Other Genes Responsible for Leaf Development

**Figure 7:**
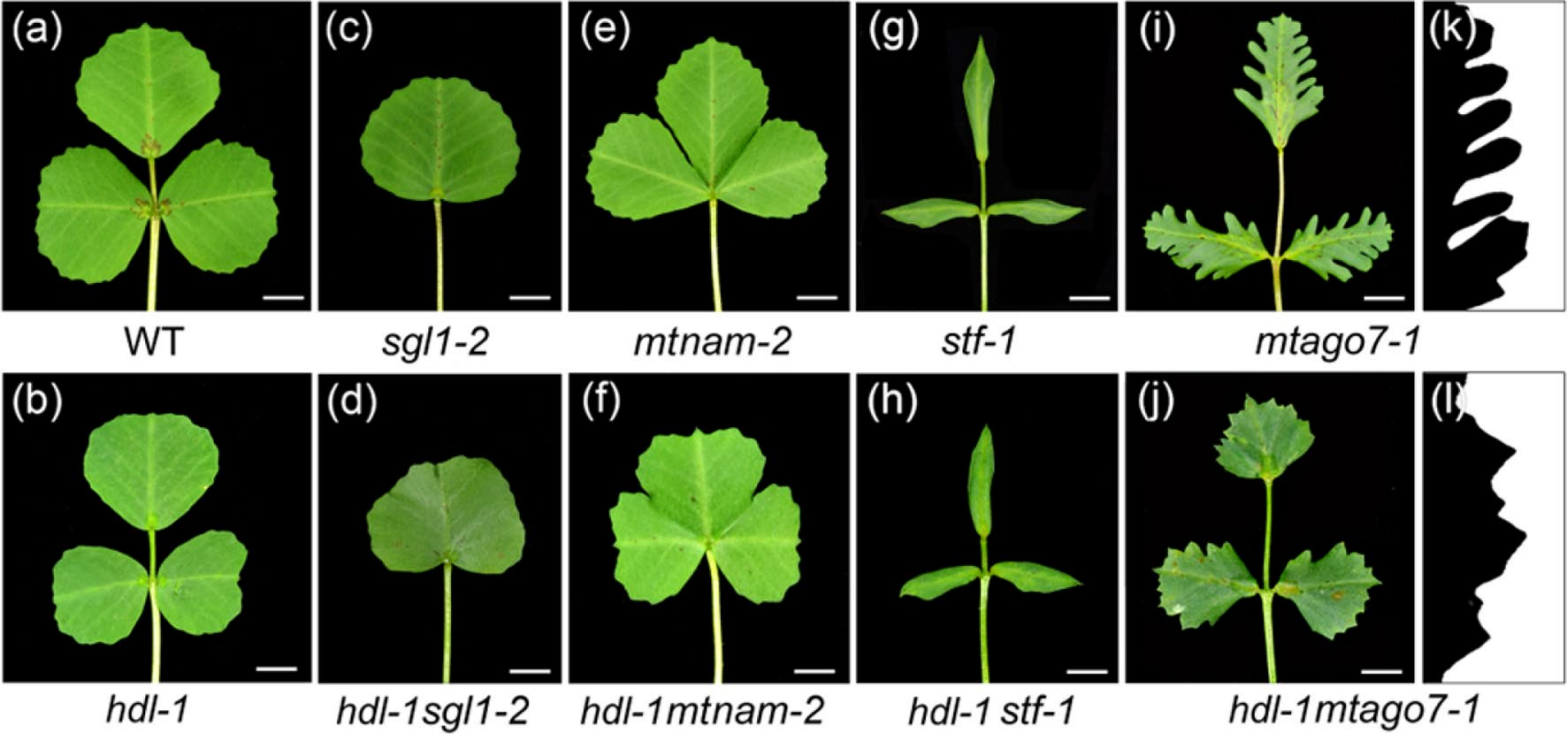
Genetic interactions between *hdl* and different leaf pattern mutants. **(A–J)** Leaf phenotype of WT **(A)**, *hdl-1* **(B)**, *sgl1-2* **(C)**, *hdl-1 sgl1-2* **(D)**, *mtnam-2* **(E)**, *hdl-1 mtnam-2* **(F)**, *stf-1* **(G)**, *hdl-1 stf-1* **(H)**, *mtago7-1* **(I)**, and *hdl-1 mtago7-1* **(J)**. Bar, 5 mm. **(K and L)** Leaf margin phenotype of *mtago7-1* **(K)** and *hdl-1 mtago7-1* **(L)**.

To investigate the possible role of HDL in leaf patterning, the *hdl-1* mutant was crossed with the leaf pattern mutants. *SGL1* is the *M. truncatula* ortholog of pea *UNIFOLIATE*; the adult leaves of LOF *sgl1* mutants are simple rather than compound (Wang et al., 2008). Double *hdl-1* s*gl1-2*mutant plants also formed simple leaves (**Figures 7A–D**), indicating that the LOF of *HDL* had no effect on leaf form if *SGL1* is disabled. The product of *MtNAM* (a member of the angiosperm *NAM/CUC* gene family) is involved in the development of lateral organ boundaries (Cheng et al., 2012). The salved offspring of an *mtnam-2*/+ heterozygote included around a quarter (presumed mutant homozygotes) in which the cotyledons were fused together. When a population of 385 salved offspring of the double heterozygote *mtnam-2/*+/*hdl-1/*+ was screened, only one double-mutant homozygote was recovered probably due to the embryo lethal (**Figure S10**). All of its leaves featured fused leaflets (**Figures 7E, F**), showing that the LOF of *HDL* had no effect on lateral organ formation if *MtNAM* is disabled. Thus, although HDL is clearly involved in compound leaf patterning, this function appears to depend on the presence of both SGL1 and MtNAM. To further investigate the potential genetic interactions between *HDL* and the gene related to leaf margin development, the *hdl-1* mutant was crossed with the *stf-1* and *mtago7-1* mutants, respectively (**Figures 7G–L**). STF is a member of the WUS-related homeobox (WOX) family of transcription factors and acts to promote cell proliferation at the adaxial-abaxial junction; plants carrying the *stf* mutation develop narrow leaves lacking marginal serration (Tadege et al., 2011). The *hdl-1 stf-1* double mutant produced a similar, narrowed leaf (**Figures 7G, H**). LOF mutants for *MtAGO7*, an ortholog of *AtAGO7*, produce lobed leaves (Zhou et al., 2013). The margins of the leaves developed by the double mutant *hdl-1 mtago7-1* were less indented than those of the *mtago7-1* mutant (**Figures 7I–L**), indicating that the *TAS3* ta-siRNA/*AGO7* pathway acts antagonistically with *HDL* in elaborating leaf margin serration.

## Discussion

### Conservation and Specialization Roles of WUS Orthologs Among Different Plant Species

The products of *WUS* and of its orthologs in both antirrhinum (*ROA*) and petunia (*TER*) all exert a major influence on the maintenance of the SAM and the AM (Laux et al., 1996; Mayer et al., 1998; Hamada et al., 2000; Stuurman et al., 2002; Kieffer et al., 2006). However, there are species differences with respect to their effect on the shoot and floral meristem and on leaf morphology. In *A. thaliana*, *wus* mutants are unable to form a viable shoot meristem in the developing embryo (Laux et al., 1996; Mayer et al., 1998), the appearance of the first rosette leaf is markedly delayed (Hamada et al., 2000), no juvenile leaves are formed (Hamada et al., 2000), and the floral meristem is terminated prematurely to create a central stamen (Laux et al., 1996; Mayer et al., 1998). Shoot development in petunia *ter* mutants is terminated after the two first true leaves have been produced; in the rare cases where plants flower, fewer floral organs are formed than normal (Stuurman et al., 2002). Finally, in antirrhinum *roa* mutants, the initiation of the SAM appears normal, but its maintenance is compromised, and the plants do not flower (Kieffer et al., 2006). The effect of the LOF of *HDL* was to disrupt the development of the shoot meristem, and there was no transition into reproductive growth. As it is also the case for mutants harboring LOF alleles of *WUS* and its orthologs, the AM of *hdl* mutants was defective, resulting in the failure to form branches.

Expression pattern diversity in key domains is an important driver of functional specialization. Some variations in the transcriptional profile of *WUS* and its orthologs have been noted, especially between dicotyledonous and monocotyledonous species. *WUS* is active in both the SAM and the developing embryo of *A. thaliana* (Mayer et al., 1998); in antirrhinum, *ROA* is transcribed in the vegetative apex, inflorescences, and young floral meristems (Kieffer et al., 2006); in petunia, *TER* has a similar transcriptional profile to *WUS* at least in the vegetative apex (Stuurman et al., 2002). Both *wus* and *ter* mutants produce only leaves, with the result that they form bushy plants. In contrast, the rice gene *TAB1* is active in emerging AMs, but its transcript cannot be detected in established SAMs (Lu et al., 2015; Tanaka et al., 2015). The *tab1* mutant is unable to form tillers, but the plant is not compromised with respect to leaf development; this has been taken to suggest that TAB1 is involved in the initiation of AMs but not in SAM maintenance (Tanaka et al., 2015). Although no *wus*-type mutant has yet to be described in maize, the transcriptional behavior of the two related genes* ZmWUS1* and *ZmWUS2* was fine-tuned by specific signal (Nardmann and Werr, 2006; Je et al., 2016). The *M. truncatula HDL* gene is active in both the SAM and the AM, and the developmental defects with respect to both the SAM and the AM associated with its LOF are comparable to those expressed in both *wus* and *roa* mutants. Unlike the latter, however, and more akin to the phenotype of the rice *tab1* mutant, *hdl* mutant plants were able to produce a number of leaves; the implication is that, in *M. truncatula*, SAM functionality is only partially compromised in the *hdl* SAM, suggesting some possible genetic redundancy between HDL and the products of other *WOX* genes. *HDL* was successfully detected in the leaf and the development of the leaf margin was defective in plants of genotype *hdl*. The species differences in the transcriptional behavior of *WUS* and its orthologs imply that these genes have experienced some neo-functionalization over the course of evolution. Overall, their functional conservation appears to have been stronger in the context of AM than of SAM growth, development, and maintenance.

### *HDL* Plays a Role With MtTPL/MtTPRs at the Protein Level

WUS acts as a repressor by recruiting the corepressor TPL (Kieffer et al., 2006; Ikeda et al., 2009). TPL and TPRs are members of Gro/Tup1 family corepressors that are complicated in a wide range of processes by directly or indirectly interacting with repressive transcription factors to repress the expression of downstream target genes (Liu and Karmarkar, 2008; Causier et al., 2012). Mutation of the WUS domain and the EAR motif interferes with the repressive activity of WUS (Kieffer et al., 2006; Ikeda et al., 2009). In addition, the LOF of the WUS-TPL interaction impairs WUS function, suggesting that the recruitment of TPL in SAM is a general mechanism to repress differentiation-promoting genes in stem cells (Kieffer et al., 2006; Causier et al., 2012). In this study, deletion of each of the WUS domain and the EAR motif or both significantly blocks the repressive activity of HDL. Thus, HDL acts mainly as a transcriptional repressor that depends on its WUS domain and EAR motif. HDL also interacts with MtTPL, and such interaction is completely abolished by the mutation or deletion of the WUS domain. Moreover, the mutation or deletion of the EAR motif reduces the intensity of their interaction. Therefore, the WUS domain and the EAR motif in HDL cooperate to recruit the MtTPL into the HDL repressor complex. Besides, HDL also interacts with all of the five MtTPRs in *M. truncatula*, suggesting a complex regulatory interaction between HDL and MtTPL/MtTPRs. It has been reported that WOX family members, STF and LFL, interact with different members of the transcriptional corepressor MtTPL/MtTPRs and are involved in *M. trunctula* compound leaf and flower development as the transcriptional repressor (Zhang et al., 2014; Niu et al., 2015). *STF* is expressed at the adaxial-abaxial layer in leaf primordia and *LFL* is expressed in the emerging petals and sepals. In contrast, *HDL* is expressed in SAM and leaf primordia. It is possible that the diverse biological functions of the WOX-MtTPL/MtTPRs complexes on *M. trunctula* development largely depend on the expression pattern or function of WOX, whereas MtTPL/MtTPRs only act as a partner or mediator.

### HDL Is Involved in Regulation of Auxin Response in Compound Leaf Morphogenesis

Plant hormones are an important regulatory component of meristem cell maintenance and differentiation. The LOF of *HDL* generated a number of abnormalities in leaf morphology, including an altered structure of the compound leaf and a different appearance of the leaf margin, which are changes that resemble those induced by the LOF of *SLM1*. Previously, it has been shown that leaf development is influenced by SLM1-mediated auxin distribution (Zhou et al., 2011). The transcriptional profile of *SLM1* in the flattened structure at the stem apex produced by the *hdl* mutant mirrors the phenotypic similarity between the *hdl* and *slm1* mutants, although the severity of the leaf developmental defects present in *hdl-1* mutants was not as great as in the *slm1 *mutant. By implication, these defects likely arose, at least in part, from a disordering of SLM1-dependent auxin gradients. The product of *STF* is known to regulate leaf growth through its control over auxin levels (Tadege et al., 2011). The behavior of *hdl-1 stf-1* double mutants supports the proposition that auxin is involved in leaf formation. Given that the margin of the leaves formed by the *stf* mutant is smooth and that *stf* is genetically epistatic to *hdl* in leaf margin formation, it is plausible to suggest that the defects in leaf development displayed by *hdl* mutant plants are related to problems in maintaining auxin homeostasis.

Some functional diversification of *WOX* genes has been observed in cross-species comparisons. In *A. thaliana*, SAM maintenance is regulated by WUS, whereas *WOX4* is not transcribed in the SAM. However, in rice, *WOX4* is transcribed in both the meristem and leaf primordia, so its product is likely involved in maintaining the meristem and regulating leaf development (Ohmori et al., 2013; Yasui et al., 2018). However, the product of the *WUS* ortholog *TAB1* participates in AM formation but not in SAM maintenance. Overall, the present data have provided novel information regarding the function of the WUS ortholog in compound leaf morphogenesis in *M. truncatula*, which underlines how the *WUS* gene family has diversified during the course of speciation.

## Conclusion

In this paper, we reported the studies of the regulatory mechanism of *HDL* in compound leaf morphogenesis in *M. truncatula*. HDL is the ortholog of *A. thaliana* WUS and plays conserved roles in the maintenance of SAM and AM. LOF in *HDL* results not only in the compromised SAM and AM but also in the altered compound leaf patterning and leaf margin formation. Based on the molecular and genetic evidence, we find that the expression pattern of *SLM1*/*PIN1*, which regulates auxin activity gradients, was impaired. Moreover, HDL positively regulates auxin response in leaves through the recruitment of MtTPL/MtTPRs into the HDL repressor complex. This study expands our knowledge about the conservation and specialization roles of WUS orthologs among different plant species, especially the regulation of auxin response by *HDL* in compound leaf morphogenesis.

## Data Availability

All datasets generated for this study are included in the manuscript/supplementary files.

## Author Contributions

HW, LuH, and CZ conceived the study and designed the experiments. HW, YX, LiH, XZ, XW, JZ, LuH, and ZM performed the experiments. ZD, Z-YW, RL, QY, FK, and CZ analyzed the data, provided the critical discussion on the work, and edited the manuscript. HW and CZ wrote the article.

## Funding

This work was supported by grants from the Ministry of Science and Technology of China (2015CB943500), the Shandong Province Natural Science Foundation (ZR2018ZC0334 and ZR2019MC013), the National Natural Science Foundation of China (projects 31671507 and 31371235), and the 1000-Talents Plan from China for Young Researchers.

## Conflict of Interest Statement

The authors declare that the research was conducted in the absence of any commercial or financial relationships that could be construed as a potential conflict of interest.
