# Supplementary material for: HEADLESS Regulates Auxin Response and Compound Leaf Morphogenesis in Medicago truncatula
Source: Front Plant Sci. 2019 Aug 16;10:1024. doi: 10.3389/fpls.2019.01024 (PMC6707262; doi:10.3389/fpls.2019.01024)
Supplement: Supplementary file 1 [file Table_1.doc]

**SUPPORTING INFORMATION**

**
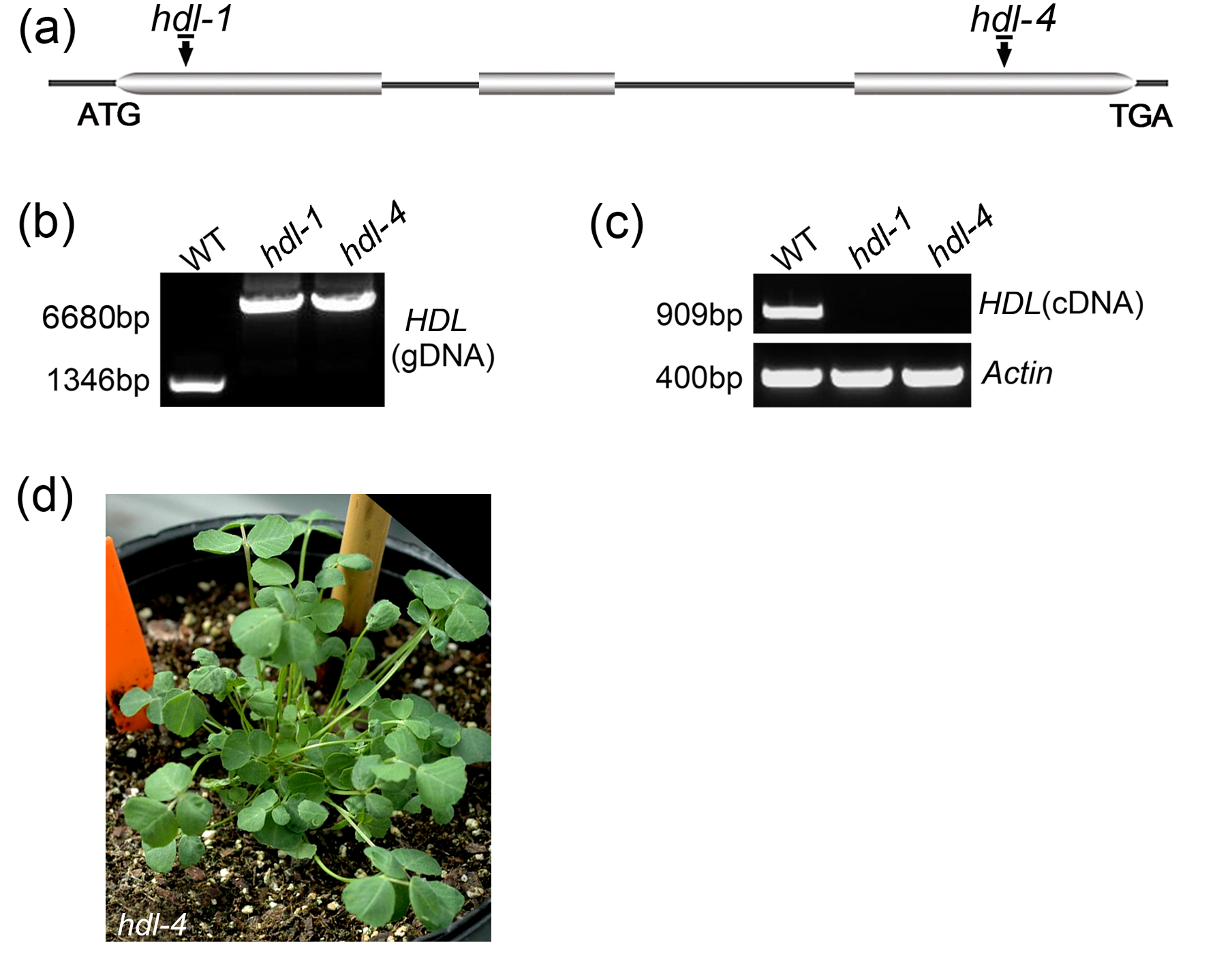
**

**Figure S1.** Molecular characterization of *HDL* in *M.truncatula*. (a) Schematic representation of the gene structure of *HDL*. The positions of the ATG start and TGA stop codons are shown. Vertical arrows mark the *Tnt1* insertion sites in the mutant lines. (b) PCR amplification of *HDL* genomic sequence from the wild type and *hdl* mutants. A single *Tnt1* insertion (~5.3 kb) was detected in *hdl-1* and *hdl-4*, respectively. (c) RT-PCR amplification of *HDL* transcripts in the wild type and *hdl* mutants. *HDL* expression was not detected in the mutants. *Actin* was used as a loading control. (d) Seven-week-old plants of the *hdl-4*.

**
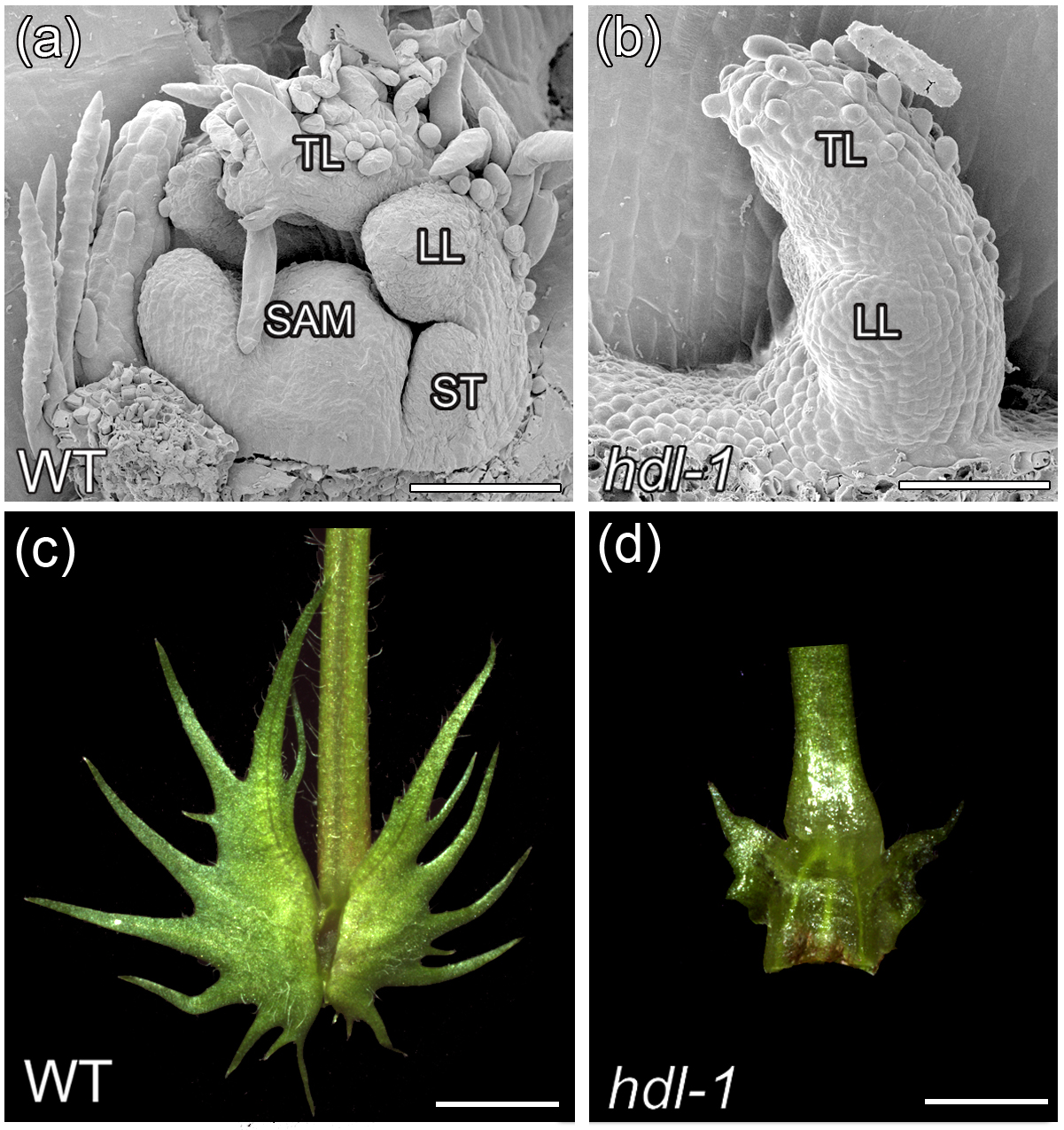
**

**Figure S2.** *hdl-1* mutant shows defects in leaf primordia and stipule development.

(a,b) SEM images of SAM and leaf primordia in the wild type (a) and *hdl-1* (b). SAM, shoot apical meristem; TL, terminal leaflet primordium; LL, lateral leaflet primordium; ST, stipule primordium. Bars in (a,b) = 50 µm.

(c,d) The *hdl-1* mutant shows defects in stipules development. (c) The wild type stipules are serrated with multiple serrations. (d) The *hdl-1* mutant stipules are less serrated and reduced in area. Bars in (c,d) = 2 mm.


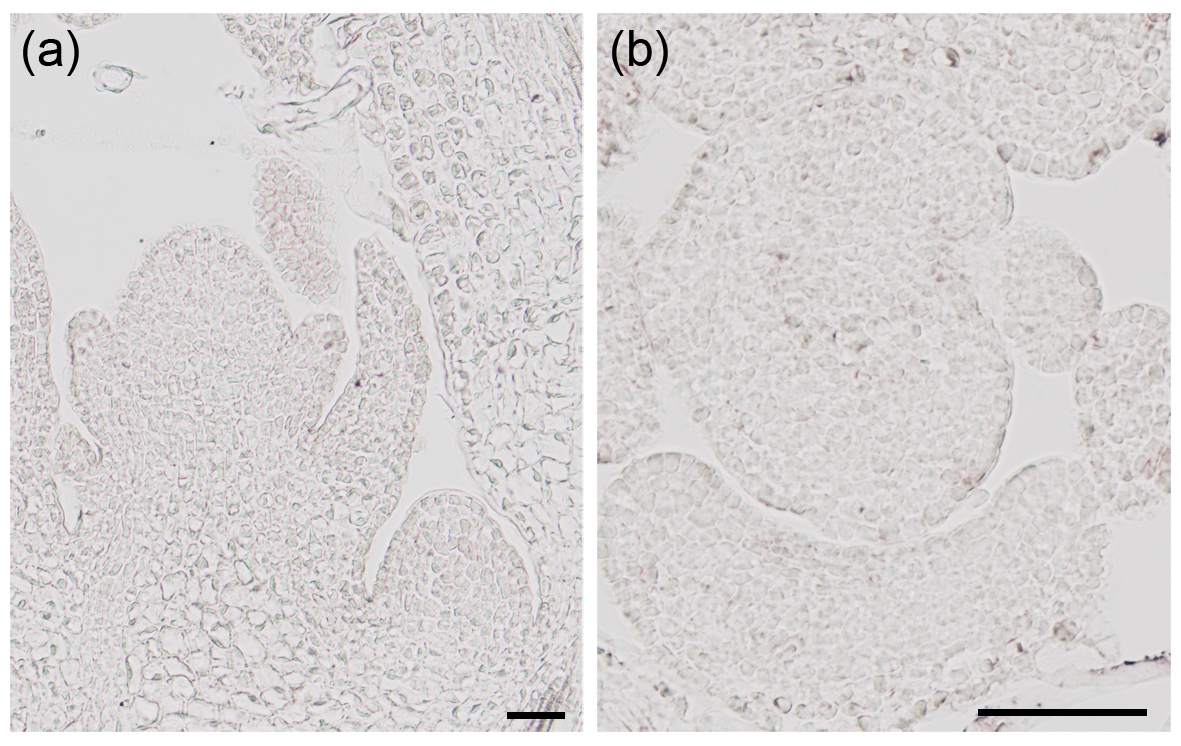


**Figure S3.** The sense probe of *HDL* was hybridized and used as the control.

(a) *HDL* sense probe in longitudinal section of the SAM. (b) *HDL* sense probe in transverse section of the SAM. Bars = 50 µm.


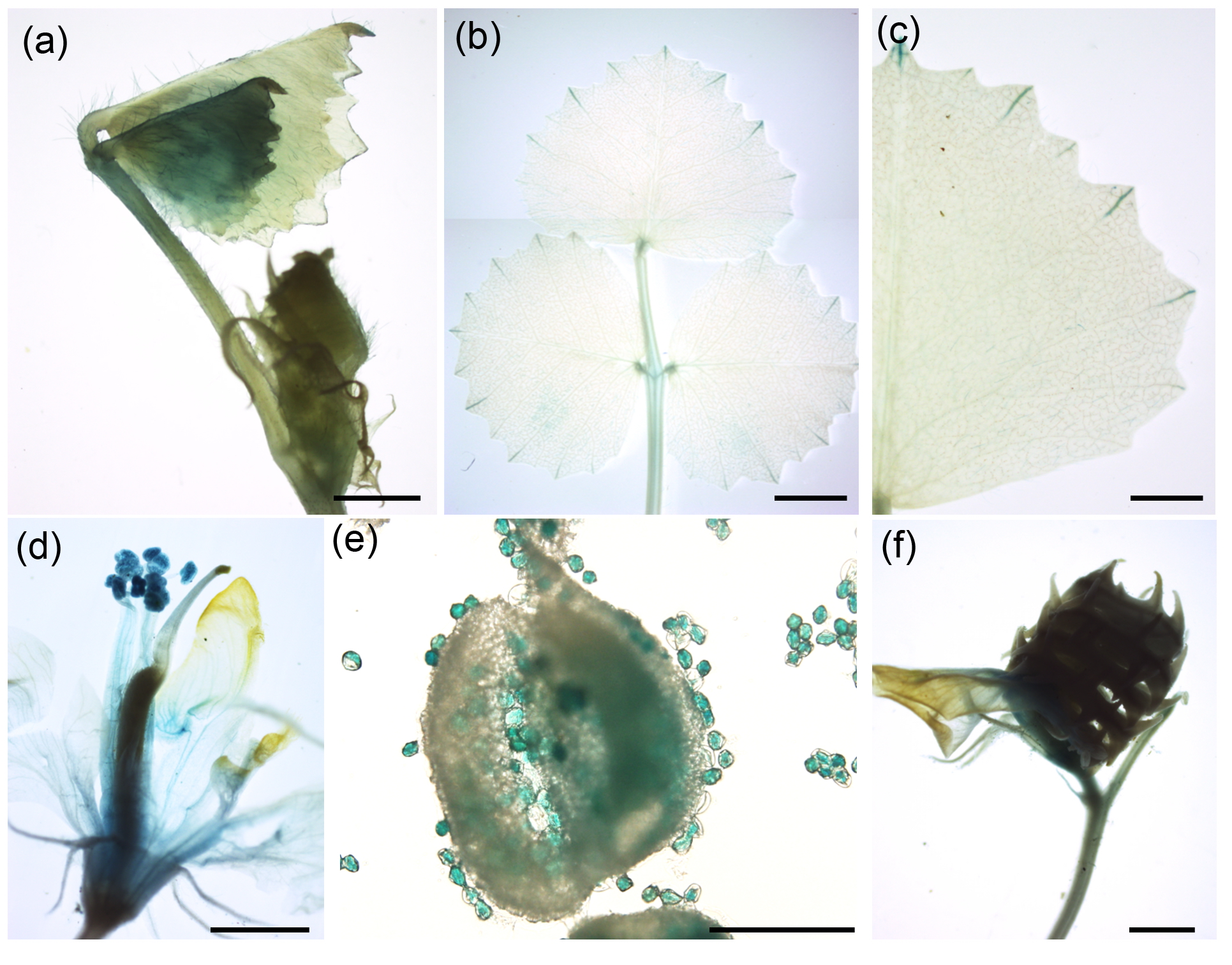


**Figure S4.** *proHDL:GUS* fusion studies of *HDL* expression in second transgenic line.

GUS histochemical staining was detected in unexpanded leaf (a), fully expanded leaf (b), leaf margin serrations (c), flower (d), anther and pollen (e), and seed pods (f). Bars, (a) 2 mm; (b-c,f) 5 mm; (d) 1 mm;(e) 50 µm.


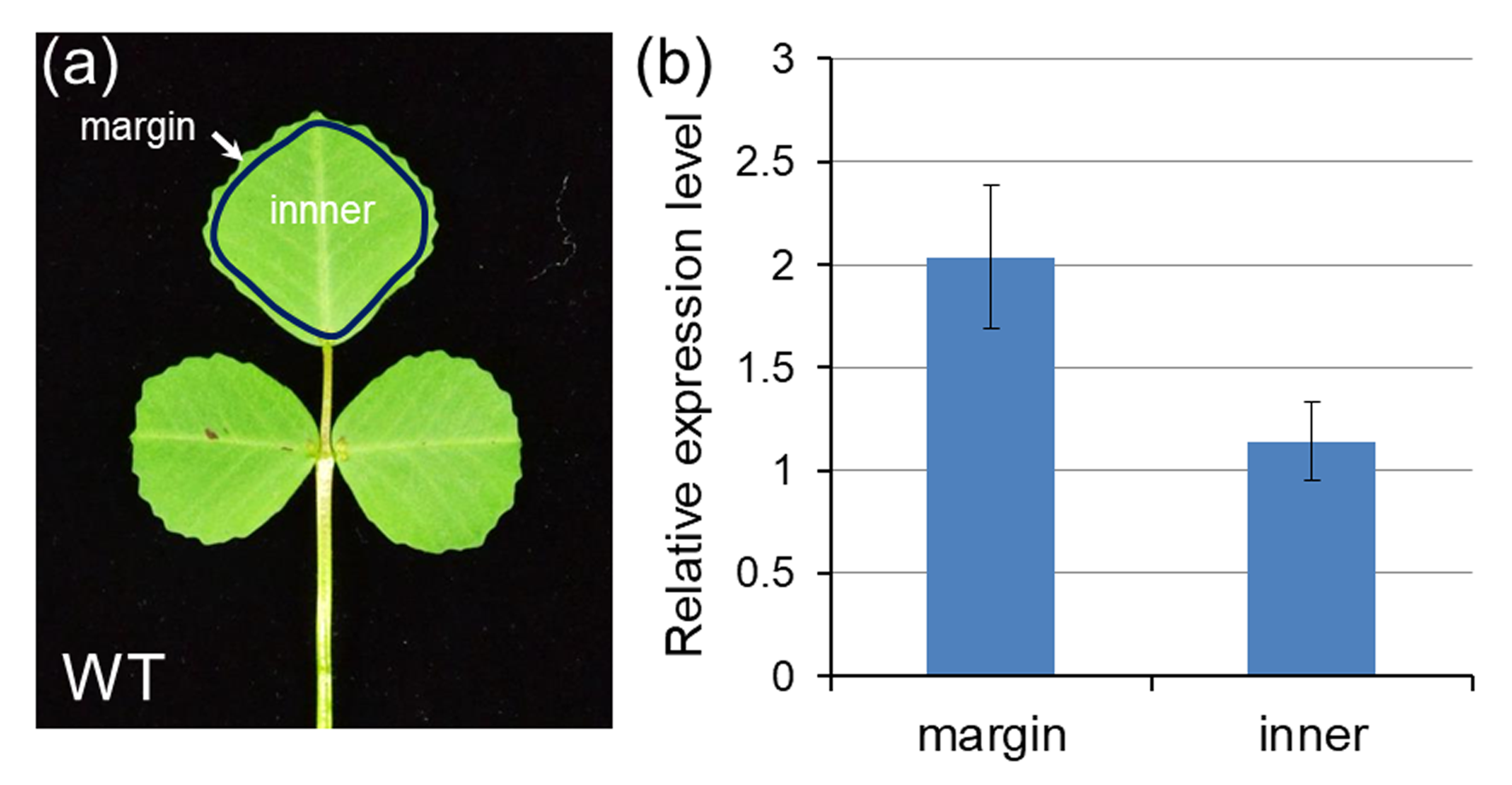


Figure S5. Transcript levels of *HDL* in marginal and inner regions of leaflets in wild-type. Values are the means and SD of three biological replicates. *MtUQI* was used as the control.


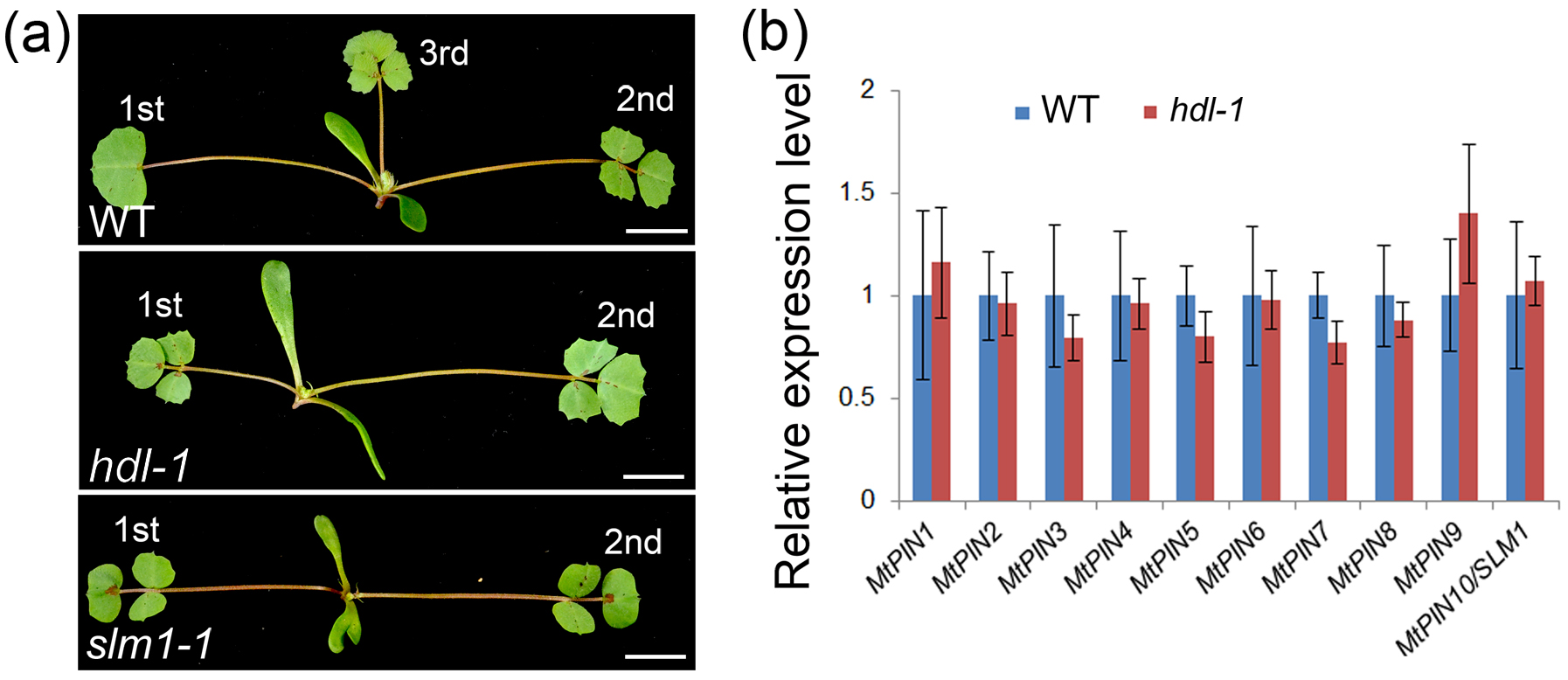


**Figure S6.** The leaf development and *MtPINs* expression in *hdl-1* mutant.

(a) Two-week-old plants of the wild type, *hdl-1*, and *slm1-1*. The juvenile leaf is the simple leaf, whose formation is defective in *hdl-1* and *slm1-1*. Bars = 1 cm.

(b) Transcript levels of all *MtPINs* in wild type and *hdl-1* mutant*.* Values are the means and SD of three biological replicates. *MtUQI* was used as the control.


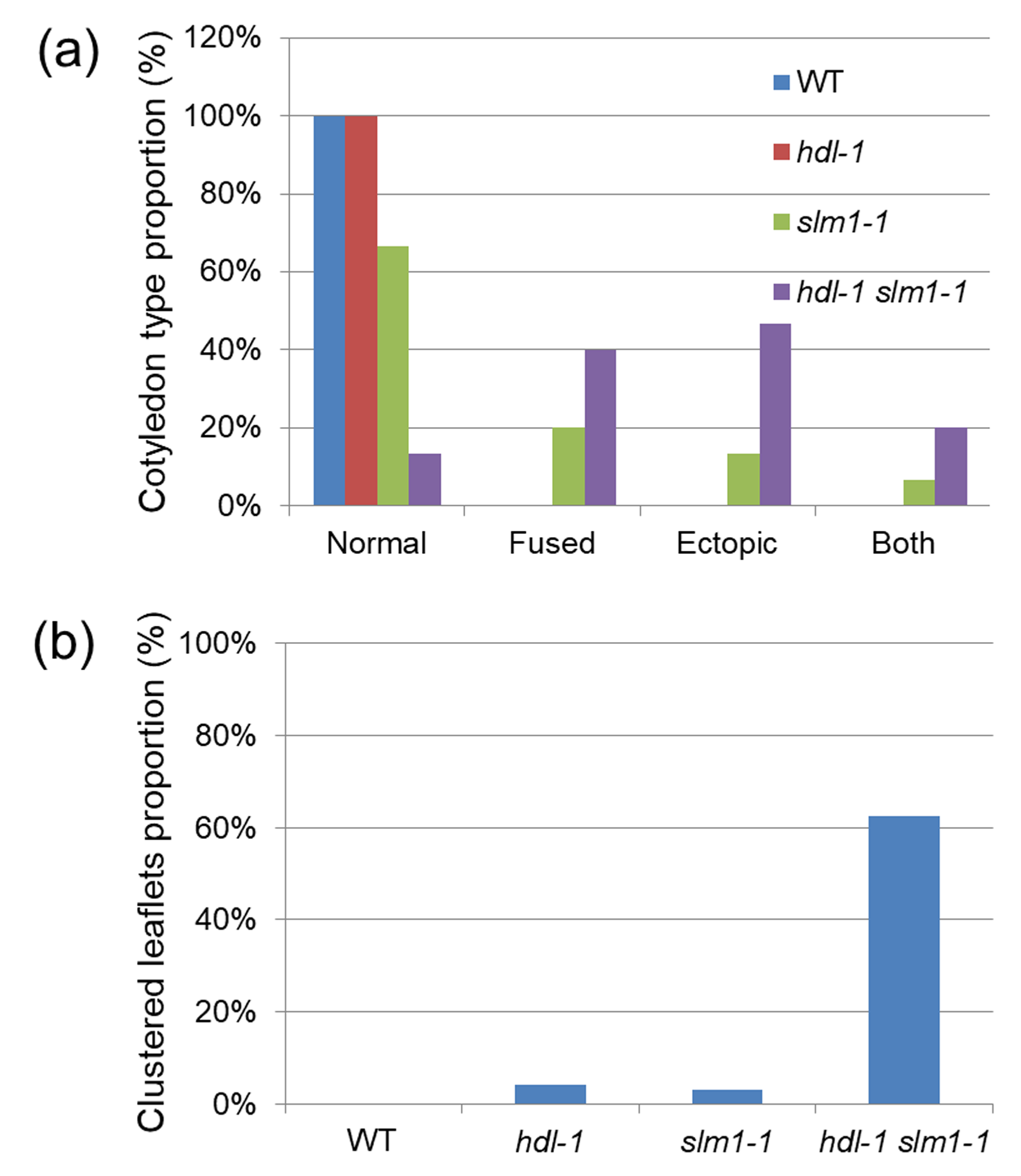


**Figure S7.** The type proportion of cotyledon and clustered leaflets in the WT, *hdl-1*, *slm1-1* and *hdl-1 slm1-1*. (a) The cotyledon type proportion in the WT, *hdl-1*, *slm1-1* and *hdl-1 slm1-1* (n=12).

(b) The clustered leaflets proportion in the WT, *hdl-1*, *slm1-1* and *hdl-1 slm1-1* (n=30).


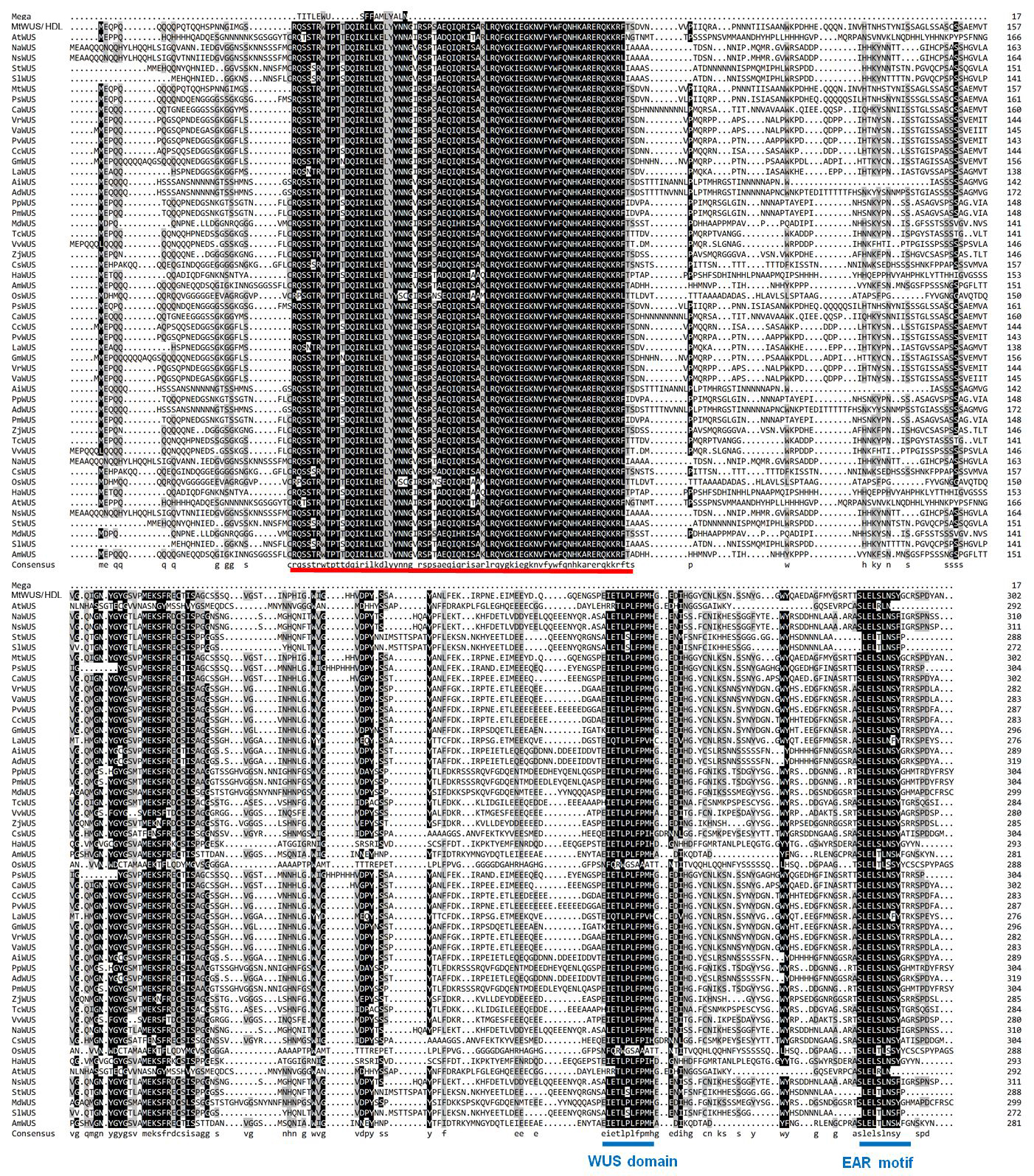


**Figure S8.** Multiple Protein Sequence Alignment of HDL and Orthologs of WUS.

Multiple protein sequence alignment of HDL and WUS orthologs among different species using DNAMAN. The homeodomain (HD) region is marked in red line. The WUS domain and EAR motif is marked in blue lines.


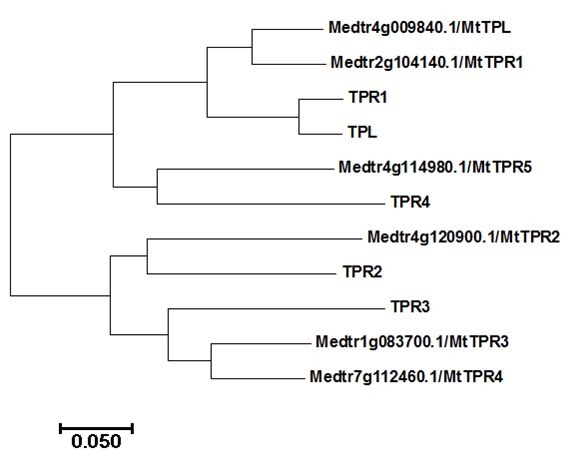


**Figure S9.** Phylogenetic analysis of TPL/TPRs family in *Arabidopsis* and *M. truncatula*. Phylogenetic analysis of *Arabidopsis* and *M. truncatula* TPL/TPRs family members using full-length amino acid sequences. The phylogenetic tree was constructed using MEGA7 by the Neighbor-Joining method with bootstrap replication of 1, 000 times.


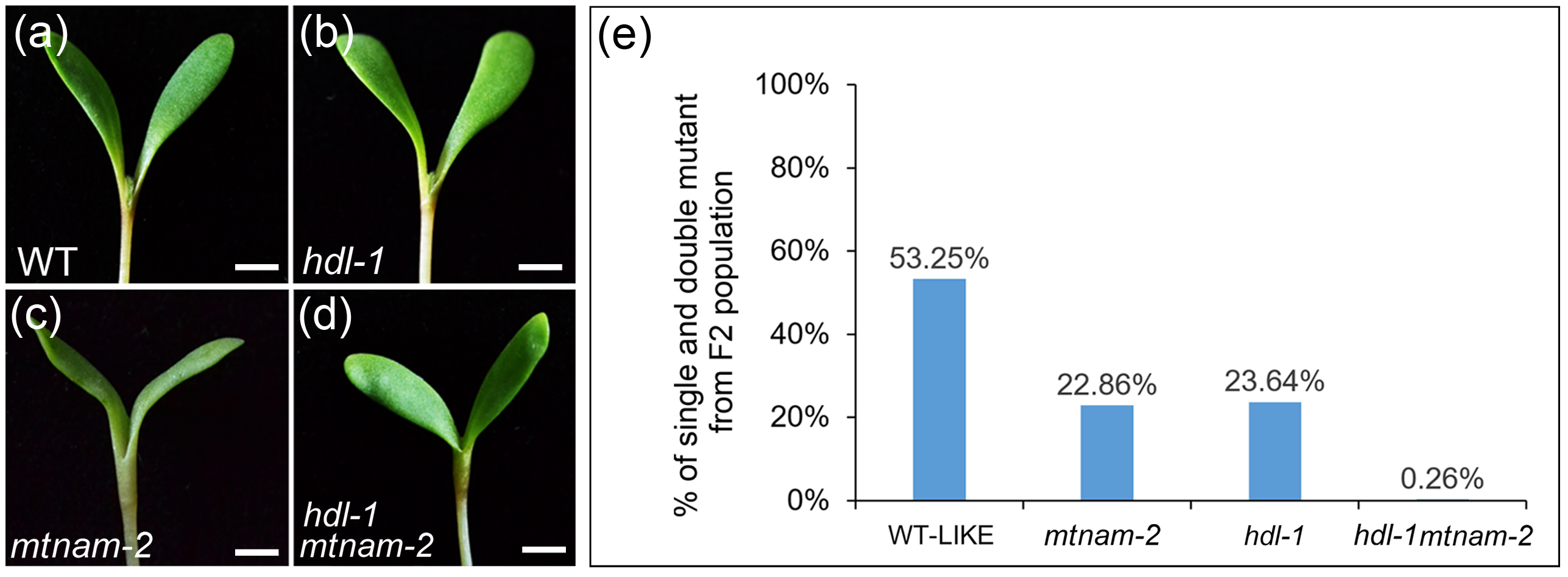


**Figure S10.** Genetic interaction between *hdl* and *mtnam*.

(a-d) Five-day-old seedlings of the wild type (a), *hdl-1* (b), *mtnam-2* (c), and *hdl-1 mtnam-2* (d). Bars in (a-d) = 5mm.

(e) Percentage of wild-type-like and mutant plants obtained from the F2 segregation population of *hdl-1*(+/-) *nam-2*(+/-).

**Table S1.** Phenotypic characterization of leaves in the 8-week-old wild type, *hdl-1*, and *slm1-1* plants.

| **Genotype** | **Terminal Leaflet Length (cm)** | **Terminal Leaflet Width (cm)** | **Ratio of  Length / Width** | **Lateral Leaflet Length (cm)** | **Lateral Leaflet Width (cm)** | **Ratio of Length / Width** |
| --- | --- | --- | --- | --- | --- | --- |
| Wild type | 2.39±0.09 | 1.92±0.12 | 1.24±0.06 | 2.06±0.08 | 1.67±0.09 | 1.23±0.08 |
| *hdl-1* | 1.81±0.18^***^ | 2.25±0.19^***^ | 0.81±0.06^***^ | 1.48±0.15^***^ | 1.80±0.15^**^ | 0.82±0.05^***^ |
| *slm1-1* | 1.80±0.14^***^ | 2.08±0.10^**^ | 0.86±0.05^**^ | 1.65±0.10^***^ | 1.93±0.12^***^ | 0.85±0.02^**^ |

Numbers are presented as mean±SD. The number of observations in each mean is 21. **P < 0.01, ***P < 0.001.

**Table S2.** Primers used in this study.

| Primer | Sequence | Application |
| --- | --- | --- |
| HDL-CDS-F | CACCATGGAACAGCCTCAACAACAACAAC | For cloning of the *HDL* full length CDS |
| HDL-CDS-R | ATTAGCATAATCTGGTGACCTACAGC |
| HDL-Prom-F | CACCTTTTTAGTAAAAATGCATCTCGCAC | For cloning of the *HDL* promoter |
| HDL-Prom-R | GTTTTTGTTGGACTGAAAACAAGAA |
| HDL-qRT-F | GATGCTGGATTCATGTATGGTTCTC | For qRT-PCR analysis of *HDL* |
| HDL-qRT-R | TGACCTACAGCCGTAAGAGTTGAG |
| HDL-Prob-F | ATGGAACAGCCTCAACAACAACAAC | For cloning of the *HDL* probe |
| HDL-Prob-R | GGTATGAACATTAATGTTTTG |
| HDL-mEAR-F | CACCATGGAACAGCCTCAACAACAACAAC | For mutation the EAR motif of HDL |
| HDL-mEAR-R | TTAATTAGCATAATCTGGTGACCTACAGCCGTAA GAGTTTGCGCTTGCTTCCAAGGAAGTAGTACG |
| HDL-△EAR-F | CACCATGGAACAGCCTCAACAACAACAAC | For deletion the EAR motif of HDL |
| HDL-△EAR-R | TTAATTAGCATAATCTGGTGACCTACAGCCGTAA CGAGAACCATACATGAATCCAGC |
| HDL-mWUS-F | GAAACCGCACCTGCATTCCCTATGCATGGTG | For mutation the WUS domain of HDL |
| HDL-mWUS-R | AGGGAATGCAGGTGCGGTTTCAATTTCAGGTG |
| HDL-△WUS-F | ACCTGAAATTGGTGAAGACATTCATGGTGGC | For deletion the WUS domain of HDL |
| HDL-△WUS-R | TGTCTTCACCAATTTCAGGTGAACCATTTTC |
| HDL-△WUS-△EAR-F | CACCATGGAACAGCCTCAACAACAACAAC | For deletion of both the WUS domain and EAR motif of HDL |
| HDL-△WUS-△EAR-R | TCAAATTTCAGGTGAACCATTTTCTTG |
| MtTPL-F | CACCATGTCATCTCTGAGTAGGGAATTG | For cloning of the N-terminal of MtTPL |
| MtTPL-R | TCACGTGACAGTCTTCGGCAATGGC |
| MtTPL-Prob-F | GCAGCATCAACTATGTAAGAATCC | For cloning of the *MtTPL* probe |
| MtTPL-Prob-R | TTCGGCAATGGCAAGTCATCAGGT |
| MtTPR1-F | CACCTATTCACCAAAATCCGAGGTCAAAC | For cloning of the N-terminal of MtTPR1 |
| MtTPR1-R | TCAATGACCATGACCCGGAAATGTC |
| MtTPR1-Prob-F | CGAAGTATGGTGATACGAAGTCTG | For cloning of the *MtTPR1* probe |
| MtTPR1-Prob-R | GACATTCCTATTGGTCTTGTTCTC |
| MtTPR2-F | CACCATGACATCTTTGAGTAGAGAATTGGTG | For cloning of the N-terminal of MtTPR2 |
| MtTPR2-R | TCAGCTTGTCAATGTTGATCCTTG |
| MtTPR3-F | CACCATGACTTCGTTGAGTAGAGAATTGG | For cloning of the N-terminal of MtTPR3 |
| MtTPR3-R | TCAGCTTGTCACAGAGGATCCTTG |
| MtTPR4-F | CACCATGACTTCTTTGAGCAGAGAATTGG | For cloning of the N-terminal of MtTPR4 |
| MtTPR4-R | TCACACAGAGGACCCTTGATGC |
| MtTPR5-F | CACCATGTCTTCGTTAAGCAGAGAACTCG | For cloning of the N-terminal of MtTPR5 |
| MtTPR5-R | TCACACAGCAGTCTTGGGCAAGTC |
| MtPIN1-qRT-F | AGACCGTCAAACTTCGAAGAGAAC | For qRT-PCR analysis of *MtPIN1* |
| MtPIN1-qRT-R | AGAGTTAACAGTCTGTGCCGATGT |
| MtPIN2-qRT-F | CCTCCTCCAAATCCTATGCTTTC | For qRT-PCR analysis of *MtPIN2* |
| MtPIN2-qRT-R | CCGCCCATGCTATCTTTCTTC |
| MtPIN3-qRT-F | AGTTATGGCAGCAGCTTCTATCG | For qRT-PCR analysis of *MtPIN3* |
| MtPIN3-qRT-R | GTGCAGCCTGAACAATAGCTACA |
| MtPIN4-qRT-F | CTGATGTGTTTGGTGGACATGAC | For qRT-PCR analysis of *MtPIN4* |
| MtPIN4-qRT-R | TTCTCTATGACCCTCCACTTTTCC |
| MtPIN5-qRT-F | CGAGCTTCAAATCTGACGAATG | For qRT-PCR analysis of *MtPIN5* |
| MtPIN5-qRT-R | CCCTCTTGGAGTTGGATTCCT |
| MtPIN6-qRT-F | CGCTCACAACGCAATCAGAA | For qRT-PCR analysis of *MtTPR6* |
| MtPIN6-qRT-R | CGTCGGATTCGGACTTTGA |
| MtPIN7-qRT-F | TCCGTGGAGTTCTCTTACATGTTG | For qRT-PCR analysis of *MtPIN7* |
| MtPIN7-qRT-R | GGCAAACACAAAGGGAACGATA |
| MtPIN8-qRT-F | GTTGTCCAATGGAGGTCTTGGT | For qRT-PCR analysis of *MtPIN8* |
| MtPIN8-qRT-R | CCCACATGCTATGATGCTTGA |
| MtPIN9-qRT-F | ACTGCATGCCGAGGTTCTTAG | For qRT-PCR analysis of *MtPIN9* |
| MtPIN9-qRT-R | CGGTAGCGAAACAATCATTCC |
| MtPIN10-qRT-F | AGGACCAGTGCTATCCAAACTTG | For qRT-PCR analysis of *MtPIN10* |
| MtPIN10-qRT-R | TTCACCTTGAGATCCAGCTTTAGG |
